# Melatonin enhances KCl salinity tolerance by maintaining K^+^ homeostasis in *Malus hupehensis*

**DOI:** 10.1111/pbi.14129

**Published:** 2023-07-19

**Authors:** Zhijuan Sun, Jianyu Li, Dianming Guo, Tianchao Wang, Yike Tian, Changqing Ma, Xiaoli Liu, Caihong Wang, Xiaodong Zheng

**Affiliations:** ^1^ College of Horticulture Qingdao Agricultural University Qingdao China; ^2^ College of Life Science Qingdao Agricultural University Qingdao China; ^3^ Engineering Laboratory of Genetic Improvement of Horticultural Crops of Shandong Province Qingdao China

**Keywords:** *Malus hupehensis*, Melatonin, KCl stress, WRKY53, K^+^ homeostasis

## Abstract

Large amounts of potash fertilizer are often applied to apple (*Malus domestica*) orchards to enhance fruit quality and yields, but this treatment aggravates KCl‐based salinity stress. Melatonin (MT) is involved in a variety of abiotic stress responses in plants. However, its role in KCl stress tolerance is still unknown. In the present study, we determined that an appropriate concentration (100 μm) of MT significantly alleviated KCl stress in *Malus hupehensis* by enhancing K^+^ efflux out of cells and compartmentalizing K^+^ in vacuoles. Transcriptome deep‐sequencing analysis identified the core transcription factor gene *MdWRKY53*, whose expression responded to both KCl and MT treatment. Overexpressing *MdWRKY53* enhanced KCl tolerance in transgenic apple plants by increasing K^+^ efflux and K^+^ compartmentalization. Subsequently, we characterized the transporter genes *MdGORK1* and *MdNHX2* as downstream targets of MdWRKY53 by ChIP‐seq. MdGORK1 localized to the plasma membrane and enhanced K^+^ efflux to increase KCl tolerance in transgenic apple plants. Moreover, overexpressing *MdNHX2* enhanced the KCl tolerance of transgenic apple plants/callus by compartmentalizing K^+^ into the vacuole. RT–qPCR and LUC activity analyses indicated that MdWRKY53 binds to the promoters of *MdGORK1* and *MdNHX2* and induces their transcription. Taken together, our findings reveal that the MT‐WRKY53‐GORK1/NHX2‐K^+^ module regulates K^+^ homeostasis to enhance KCl stress tolerance in apple. These findings shed light on the molecular mechanism of apple response to KCl‐based salinity stress and lay the foundation for the practical application of MT in salt stress.

## Introduction

Potassium (K), an essential plant nutrient element, plays important roles in metabolic enzyme activity, signal transduction, photosynthesis and respiration (Johnson *et al*., 2022; Lefoulon, 2021; Wang and Wu, 2017). K is also a key factor determining the NaCl‐induced salt tolerance of non‐halophytes due to the core regulatory mechanism based on ‘sodium efflux and potassium absorption’ (Parida and Das, 2005; Zhu, 2000). In apple (*Malus domestica* Borkh.) orchards, potassium deficiency (–K) inhibits photosynthesis and reduces the transpiration rate, thereby decreasing fruit yields (Khachtib *et al*., 2022; Kuzin *et al*., 2020). Hence, large amounts of potassium fertilizer are widely applied to enhance production and fruit quality. However, this exogenous application leads to excess K in the soil, resulting in KCl‐induced salt stress (Zheng *et al*., 2021a). KCl stress can induce ion toxicity, osmotic stress and oxidative damage, which seriously harm the vigour of apple trees (Buvaneshwari *et al*., 2020). Therefore, strategies are needed to improve the resistance of apple rootstocks to KCl stress.

Melatonin (MT; N‐acetyl‐5‐methoxytrytamine) was first identified in plants in 1995 (Dubbels *et al*., 1995). Since then, it has been widely reported to improve plant tolerance against a variety of abiotic stresses, such as cold stress, drought stress, alkaline stress and salt stress (Arnao and Hernandez‐Ruiz, 2019; Gong *et al*., 2017). MT was recently reported to be involved in plant responses to ionic stress. MT enhances plant tolerance to iron deficiency by scavenging reactive oxygen species (ROS) in apples (Nawaz *et al*., 2018; Zheng *et al*., 2021b). Exogenous MT treatment alleviated cadmium uptake and toxicity in apple rootstocks (He *et al*., 2020). The role of MT in wheat (*Triticum aestivum*) experiencing K deficiency has also been explored. An appropriate concentration (50 μm) of MT promoted tolerance to K deficiency by regulating the activity of the high‐affinity K^+^ transporter HAK1 and its upstream transcription factor NAC71 (Li *et al*., 2021b). However, the precise role of MT in KCl stress remains to be elucidated.

MT functions in plant development and abiotic stress responses via its signal transduction pathways. MT‐regulated stomatal closure depends on the H_2_O_2_ and Ca^2+^ signal transduction cascade mediated by its receptor CANDIDATE G‐PROTEIN COUPLED RECEPTOR 2 (CAND2, also named PHYTOMELATONIN RECEPTOR 1 [PMTR1]) (Li *et al*., 2020; Wei *et al*., 2018a). This signal transduction is followed by Mitogen‐activated protein kinase (MAPK) cascade‐mediated phosphorylation. The MAPK pathway (MPK3, MPK6, MPK KINASE 4 [MKK4], MKK5, MKK7 and MKK9) is required for MT‐mediated defence responses in plants (Lee and Back, 2016). Transcription factors receive phosphorylation signals and transmit them to downstream target genes, thus playing crucial roles in signal transduction pathways. Exogenous MT treatment enhanced plant resistance to drought stress by up‐regulating the genes encoding the transcription factors C‐REPEAT BINDING FACTORS (CBFs)/DROUGHT RESPONSE ELEMENT BINDING 1 (DREB1s), resulting in increased transcription of downstream target genes (COLD‐REGULATED 15A [*COR15A*], *RESPONSIVE TO DESICCATION 22* [*RD22*] and *KIN1*) (Shi *et al*., 2015).

WRKY transcription factors are one of the largest families of transcriptional regulators in plants (Jiang *et al*., 2017). These transcription factors were recently shown to participate in MT signal transduction pathways. Transcriptome deep sequencing (RNA‐seq) showed that the expression levels of *WRKY* transcription factor genes were different in MT‐treated plants compared with the control in cucumber (*Cucumis sativus*) (Zhang *et al*., 2014). However, whether WRKY transcription factors function in MT‐mediated responses to KCl stress requires further exploration. WRKY proteins bind to the W‐box (TTGAC) elements in the promoters of their target genes and activate or inhibit their expression to regulate stress responses (Rushton *et al*., 2010; Wani *et al*., 2021). Overexpressing *MdWRKY30* up‐regulated the expression of NaCl stress response genes and enhanced the NaCl tolerance of apple callus (Dong *et al*., 2020). The transcription factor AtWRKY33 directly regulates *POTASSIUM TRANSPORTER 2* (*AtKUP2*) to improve salt stress tolerance by maintaining cellular ion homeostasis in Arabidopsis (*Arabidopsis thaliana*) (Rajappa *et al*., 2020). Nevertheless, whether WRKY transcription factors function in the plant response to KCl stress is unclear, and their direct downstream target genes remain unknown.

The main mechanism of the plant response to KCl stress involves maintaining the balance of K^+^ in the cytoplasm. The K^+^ transport mechanism in cells is complex. On one hand, cells can absorb K ions through K^+^ transporters (KT/HAK/KUP family) (Li *et al*., 2018). On the contrary, outward‐rectifying channels mediate K^+^ release and open under depolarized membrane potentials; these include GUARD CELL OUTWARD‐RECTIFYING K (GORK) and STELAR K OUTWARD RECTIFIER (SKOR) channels (Adem *et al*., 2020; Ragel *et al*., 2019). SKOR channels are outward rectifying oscillator‐type K^+^ channels that are responsible for the long‐distance transport of K^+^ through the root xylem (Chen *et al*., 2021). The other major outward‐rectifying K^+^ channel in guard cells is GORK, which contributes to K^+^ efflux to decrease turgor pressure and induce stomatal closure (Hosy *et al*., 2003). The activation of K^+^ release through GORK is critically important for stomatal closure, which is regulated by phytohormones (Demidchik *et al*., 2010; Hosy *et al*., 2003). On the other hand, K^+^ transport between the cytoplasm and vacuoles is another important mechanism for K^+^ homeostasis in plant cells (Wang *et al*., 2021b). Na^+^(K^+^)/H^+^ antiporters (NHXs) are responsible for the exchange of Na^+^/H^+^ and K^+^/H^+^ between the cytoplasm and vacuoles (Dragwidge *et al*., 2018; Isayenkov *et al*., 2020; Karim *et al*., 2021). In Arabidopsis, AtNHX1 to AtNHX4 is located in the tonoplast, and multiple triple and quadruple *nhx* knockdown mutants show reduced growth. Exposure to NaCl improved their growth, whereas KCl was harmful to some of these knockdown mutants (Bassil *et al*., 2019), indicating the important roles of these NHXs in K^+^ homeostasis.

To date, few studies have focused on the transcriptional regulation of *GORK* and *NHX* genes. Adler *et al*. (2010) reported that the sugar beet (*Beta vulgaris*) gene *BvNHX1* is regulated by a MYB transcription factor. Moreover, the transcriptional regulation of *FrNHX1* was correlated with salt tolerance in slender red fescue (*Festuca rubra* ssp. *Litoralis*), and cDNA‐array analysis showed that the salt‐regulated transcripts included a WRKY‐type transcription factor gene, suggesting that WRKY transcription factors might regulate *NHX* genes (Diédhiou *et al*., 2009). Moreover, recent studies demonstrated that the Arabidopsis GORK K‐channel is phosphorylated by CALCIUM‐DEPENDENT PROTEIN KINASE 21 (CPK21), which is in turn activated by 14–3‐3 proteins (van Kleeff *et al*., 2018). In addition, the jasmonate‐mediated activation of GORK K channels functions via a Ca^2+^ sensor‐kinase CALCINEURIN B‐LIKE PROTEIN 1 (CBL1)–CBL‐INTERACTING PROTEIN KINASE 5 (CIPK5) complex (Förster *et al*., 2019). However, little is known about the transcriptional regulation of *GORK*.

In this study, we investigated the roles of different concentrations of MT on Chinese crab apple (*Malus hupehensis*) seedlings under KCl stress by examining ion homeostasis, osmotic balance and oxidative damage. We then identified the transcription factor gene *MdWRKY53* as a core gene that responds to both KCl and MT treatment via RNA‐seq analysis. To explore the role of *MdWRKY53* in apple under KCl stress and MT treatment, we transformed apple with this gene and analysed its regulatory mechanism by chromatin immunoprecipitation followed by sequencing (ChIP‐seq). Our results indicate that MT alleviates KCl stress by inducing K^+^ efflux outside of the cell and compartmentalizing K^+^ into vacuoles by controlling the transcriptional regulation of *MdGORK1* and *MdNHX2* via the transcription factor MdWRKY53 in apple. These findings shed light on the molecular mechanism of the response of apple to KCl stress and could facilitate the development of new strategies to enhance the KCl tolerance of apple rootstocks, which is crucial for apple production.

## Results

### Exogenous MT treatment alleviates KCl stress in apple

**Figure 1:**
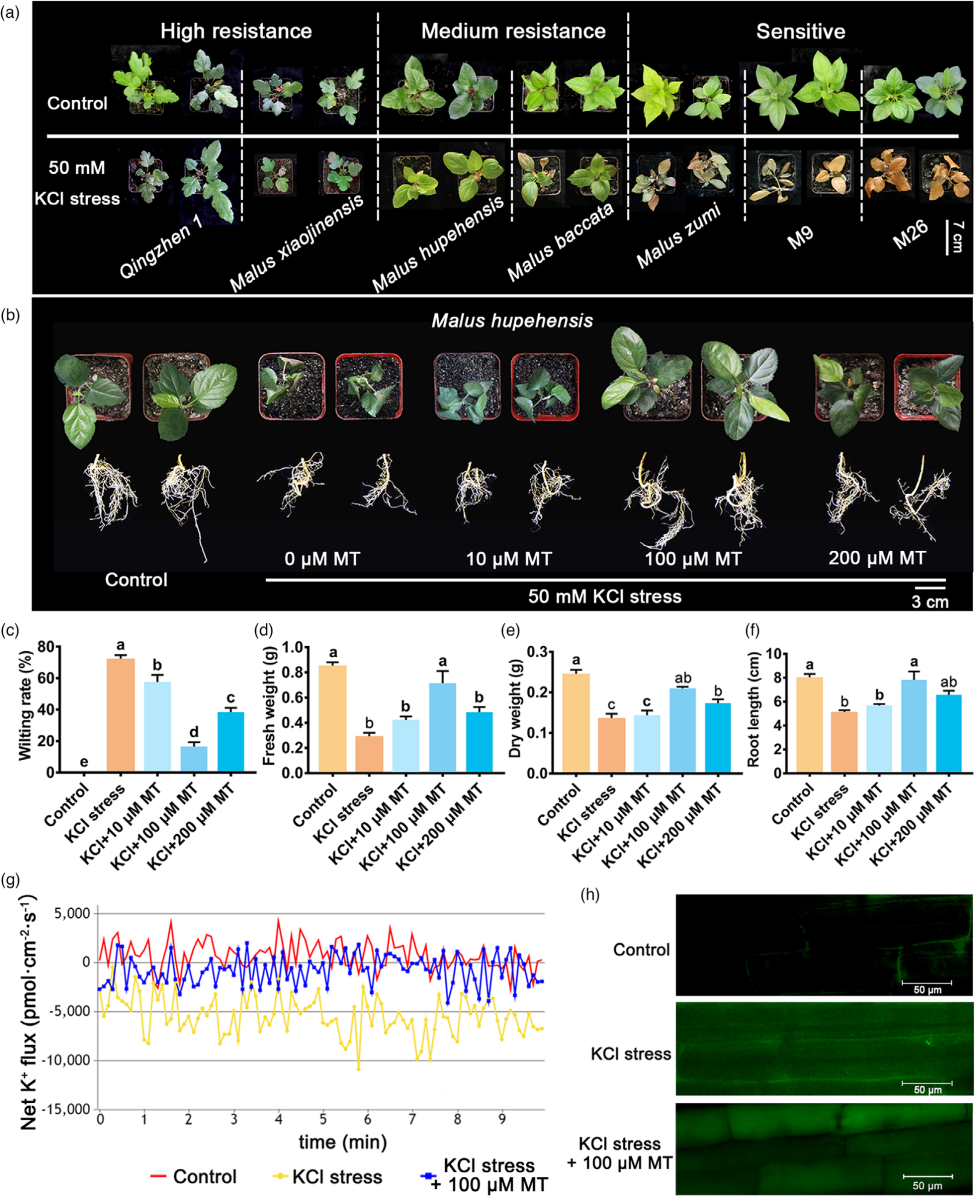
Phenotype of apple rootstocks under KCl stress and different concentrations of melatonin (MT) treatment. (a) Phenotype of seven kinds of apple rootstocks treated by 50 mm KCl stress for 20 days. Phenotype (b), the wilting rate (c), fresh weight (d), dry weight (e), root length (f) of *Malus hupehensis* seedlings with exogenous application of 0, 10, 100 and 200 μm MT under 50 mm KCl stress for 30 days. The net K^+^ flux (g) and K^+^ distribution (h) in roots of *Malus hupehensis* seedlings treated with KCl stress and exogenous 100 μm MT for 6 h. Data represent the means ± SD of triplicate experiments. Different lowercase letters indicate significant differences according to Tukey's HSD (*P* < 0.05).

In order to figure out the resistance of different apple rootstocks to KCl stress, seven widely used apple rootstocks (*Malus baccata*, *Malus xiaojinensis*, M9, M26, *Malus zumi*, *Qingzhen 1* and *Malus hupehensis*) were subjected to 50 mm KCl for 20 days. As shown in Figure 1a, *Qingzhen 1* and *Malus xiaojinensis* showed high KCl resistance, *Malus hupehensis* and *Malus baccata* showed medium resistance, while *Malus zumi*, M9 and M26 were KCl sensitive. This phenotype was also supported by physiological data including wilting rate, fresh weight and dry weight (Figure S1a–c). Therefore, *Qingzhen 1*, *Malus hupehensis*, M26 were selected to represent high‐/medium‐/low‐KCl resistance for further research.

To explore the effects of MT on KCl stress in apple plants, *Malus hupehensis* was used to screen appropriate MT concentration. As shown in Figure 1b, the *Malus hupehensis* seedlings were substantially wilted under 50 mM KCl stress for 30 days. However, exogenous MT significantly alleviated the wilting of *Malus hupehensis* seedlings. The wilting rates under 10, 100 and 200 μm MT treatment were 56.0%, 15.8%, and 37.8%, respectively, which were all markedly lower compared with the seedlings exposed to KCl stress but with no MT (71.2%) (Figure 1c). Moreover, the fresh and dry weights of seedlings experiencing KCl + MT treatment were significantly higher than those treated with KCl only (Figure 1d, e). Root growth was significantly inhibited by KCl stress, with root lengths decreasing from 7.9 to 5.0 cm. When exogenous MT was applied to these seedlings, root length under 10, 100 and 200 μm MT treatment increased to 5.6, 7.8 and 6.5 cm, respectively (Figure 1f). Of the three MT concentrations tested, 100 μm had the best effects, with the lowest wilting rate and highest fresh weight, dry weight and root length compared with 10 and 200 μm MT (Figure 1). Therefore, we selected 100 μm MT for further analysis.

The effect of 100 μm MT on *Qingzhen 1* (high resistance) and M26 (low resistance) under KCl stress were also explored. It was shown that similar to *Malus hupehensis*, exogenous 100 μm MT also substantially recovered the wilting phenotype of *Qingzhen 1* and M26 apple plants under KCl stress (Figure S1d). This phenotype was also supported by wilting rate, fresh weight and dry weight detection (Figure S1e–g). These results indicate that exogenous MT alleviates KCl stress in apple plants. *Malus hupehensis*, as a widely used rootstock in apple production with medium KCl resistance, had the largest increase in the fresh weight and dry weight after MT application compared with *Qingzhen 1* and M26 (Figure S1f, g). Thus, it was selected for the mechanism study.

### Exogenous MT relieves oxidative damage and osmotic stress induced by KCl treatment

KCl stress induced serious oxidative damage to *Malus hupehensis* seedlings, as reflected by ROS staining in roots and H_2_O_2_ and O_2_—staining in leaves. However, in the presence of 100 μm MT, the harmful effects of KCl decreased, as the staining became lighter compared to seedlings exposed only to KCl stress (Figure S2a, b). This finding was supported by the ROS fluorescence data and measurements of malondialdehyde (MDA, a marker of lipid peroxidation) levels: ROS and MDA levels significantly increased under KCl stress and decreased after exogenous MT treatment (Figure S2c,d).

The activities of the antioxidant enzymes peroxidase (POD) and catalase (CAT) decreased from 15.7 to 2.1 U/mg and 3.0 to 1.7 U/mg, respectively, after KCl stress, with no significant change in superoxide dismutase (SOD) activity. However, adding exogenous MT along with KCl significantly increased POD and CAT activities (Figure S2e–g). These results indicate that exogenous MT relieves oxidative damage induced by KCl stress by enhancing POD and CAT activities. In addition, electrolyte leakage rose 3.8‐fold after KCl treatment but decreased by 53% following the application of MT (Figure S2h). Proline contents increased 4.7‐fold in response to KCl stress and increased by 6.4 μg/g FW (fresh weight) following the application of MT (Figure S2i). The soluble sugar contents increased from 7.5 μg/g FW to 30 μg/g FW after KCl stress and decreased to 19 μg/g FW following the application of MT (Figure S2j). These results suggest that exogenous MT alleviates osmotic stress in *Malus hupehensis* induced by KCl stress by increasing proline contents.

### Effects of exogenous MT on mineral element contents and K^+^ homeostasis under KCl stress

We measured the contents of macronutrients and micronutrients in *Malus hupehensis* seedlings under KCl and MT treatment. As shown in Figure S3a, KCl stress significantly increased the contents of the macronutrients K, sodium (Na), calcium (Ca) and magnesium (Mg). When the seedlings were treated with KCl + MT, their K and Mg contents decreased significantly, while Na contents increased. The K content increased from 9.6 to 27.3 mg g^−1^ under KCl stress, while it decreased by 6.1 mg/g when exogenous MT was applied. The contents of the micronutrients iron (Fe), manganese (Mn) and copper (Cu) significantly increased under KCl stress and continued to increase after exogenous MT application (Figure S3b). We also examined the K:Na ratio, which reflects the KCl tolerance of plants. The K:Na ratio increased 2.4‐fold under KCl stress compared with the control. However, this ratio decreased by 72% after MT treatment (Figure S3c). These results indicate that exogenous MT alleviates KCl stress mainly by regulating the K:Na ratio.

We then focused on K^+^ homeostasis in cells. We examined K^+^ flux in *Malus hupehensis* seedlings under KCl stress and exogenous MT treatment. As shown in Figure 1g, the net K^+^ flux of seedlings under KCl stress was −5010.4 pmol·cm^−2^·s^−1^, which was significantly lower than that under control conditions. Under exogenous MT + KCl treatment, net K^+^ flux increased to −619.4 pmol cm^−2^ s^−1^ compared to that under KCl stress alone. These results indicated that the influx of K^+^ strongly increases under KCl stress, while exogenous MT application significantly enhanced the efflux of K^+^ under KCl stress (Figure 1g). We also examined K^+^ distribution under KCl stress and exogenous MT treatment by staining the seedlings with ION Potassium Green. We detected significantly more K^+^ in cells under KCl stress than under control conditions, particularly in the cytoplasm. Following exogenous MT treatment, we observed the distribution of K^+^ in both the cytoplasm and vacuole, particularly in vacuole (Figure 1h). These results suggest that MT compartmentalizes K^+^ into the vacuole and alters the distribution of K^+^ under KCl stress.

To confirm the K^+^ homeostasis mechanism under KCl stress and MT treatment in apple, the net K^+^ flux and K^+^ distribution in *Qingzhen 1*, *Malus hupehensis*, M26 were detected. The results showed that net K^+^ flux was highest in *Qingzhen 1*, followed by *Malus hupehensis*, and lowest in M26 under KCl stress. When 100 μm MT was applied, the net K^+^ flux were all significantly increased in three kinds of apple roots, and *Malus hupehensis* had the largest increase compared with *Qingzhen 1* and M26 (Figure S1h). These results indicated that the efflux of K^+^ was an important KCl tolerance mechanism in apple and MT application significantly enhanced the efflux of K^+^ under KCl stress. Meanwhile, we examined K^+^ distribution by staining the roots with ION K Green. It was found that K^+^ was mainly distributed in cytoplasm of *Malus hupehensis* and M26 roots under KCl stress, while K^+^ was mostly compartmentalized in vacuole of *Qingzhen 1* roots. When exogenous MT was applied, most of K^+^ was all compartmentalized into vacuole of *Qingzhen 1*, *Malus hupehensis*, M26 root cells and *Malus hupehensis* had the most substantially change among them (Figure S1i). These results suggested that vacuole K^+^ compartmentalization was another KCl tolerance mechanism in apple and MT enhanced compartmentalizes K^+^ into the vacuole under KCl stress.

### Identification of *MdWRKY53* by RNA‐seq of KCl + MT‐treated *Malus hupehensis* seedlings

**Figure 2:**
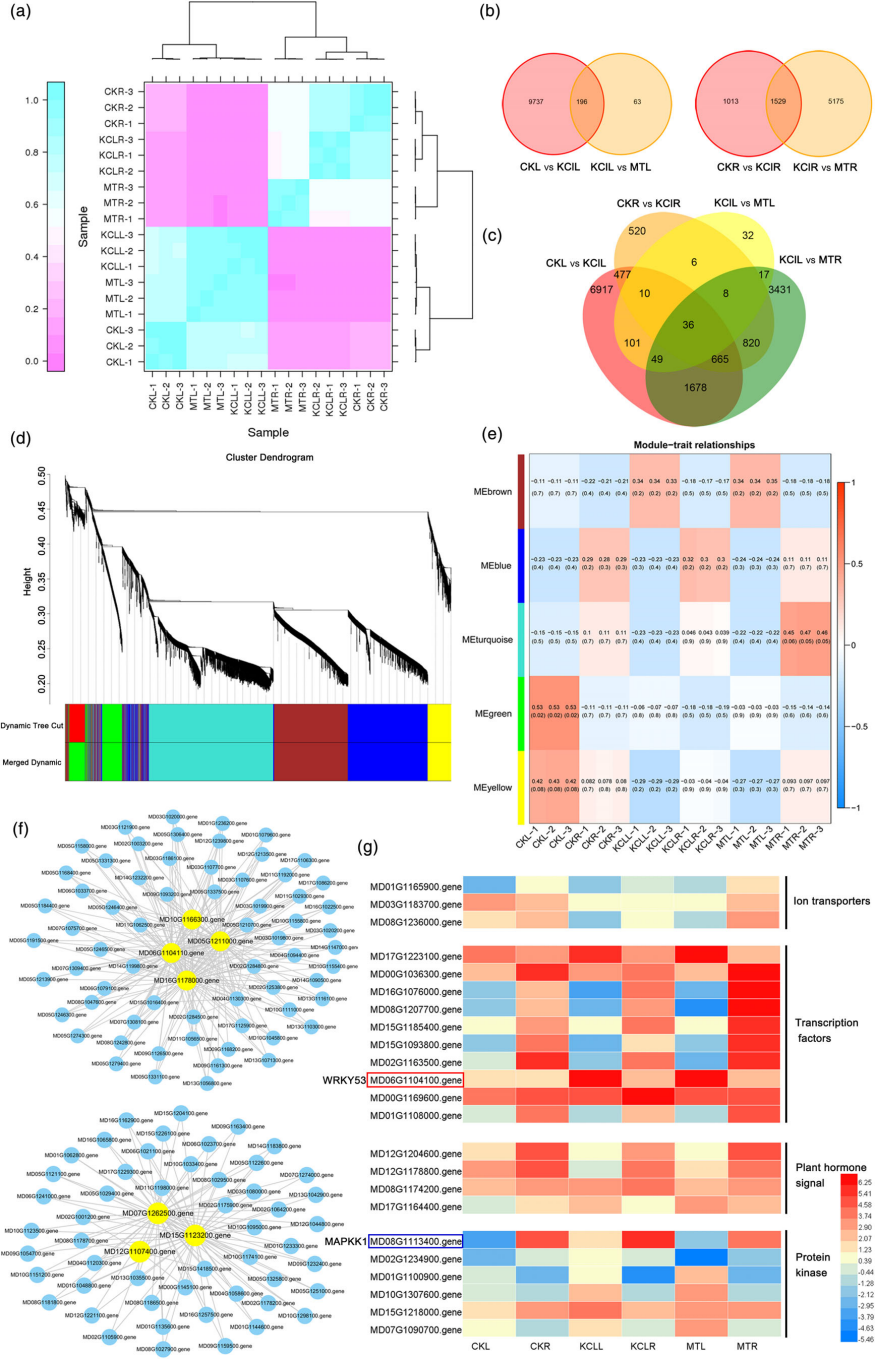
RNA sequencing (RNA‐seq) and differentially expressed genes (DEGs) analysis of apple seedlings treated with KCl stress and exogenous MT. (a) Pearson's Correlation Coefficient (R) between different replicates. (b and c) Venn diagram of co‐responding genes in leaves and roots treated with KCl stress and KCl + MT. (d) Clustering dendrogram showing five modules of co‐expressed genes based on the topological overlap in all samples. (e) Relationship between modules and different samples. The correlation value for each module–trait pair is shown from −1 to 1. The *p*‐value for each module‐trait comparison is displayed in parentheses. (f) Cytoscape of co‐expressed genes in brown and turquoise module. (g) Expression heatmap of DEGs related to ion transports, transcription factors, plant hormone signalling, and protein kinases. The letter ‘L' was stood for leaves and ‘R' was for roots. ‘CKL' represented the leaves of control group, ‘CKR' represented the roots of control group, ‘KClL' and ‘KClR' represented the leaves or roots of KCl stress group, ‘MTL' and ‘MTR' represented the leaves or roots of KCl + MT treatment group.

We analysed the leaves and roots of *Malus hupehensis* seedlings treated with KCl stress and KCl + MT for 0 and 6 h by RNA‐seq and used Pearson's correlation coefficients (R) to evaluate the correlation among biological repeats. A total of 121.82 Gb clean data was obtained, and the percentage of Q30 bases in various samples was not less than 90.83%. The comparison efficiency between reads of each sample and the apple reference genome was between 63.80% and 86.07% (Tables S1 and S2). *R* ^2^ was close to 1, reflecting a strong correlation between the replicates (Figure 2a). We then constructed a Venn diagram of the differentially expressed genes (DEGs) from pairwise comparisons, finding that the expression of 196 genes responds to both KCl and KCl + MT treatment in leaves, while 1529 responded to these treatments in roots. Only 36 DEGs overlapped between leaves and roots, representing genes that responded to KCl and MT treatment in both leaves and roots (Figure 2b,c). Gene ontology (GO) term enrichment and Kyoto encyclopedia of genes and genomes (KEGG) pathway analysis showed that the DEGs are significantly enriched in biological process terms including cellular process, metabolic process and single‐organism process; the cellular component terms membrane and membrane part, and the molecular function terms transporter activity, catalytic activity and protein binding (Figure S4a,b).

We performed weighted gene co‐expression network analysis (WGCNA) to identify key genes that function at the nodes of gene‐expression networks. We identified five modules, with the DEGs that responded to both KCl and MT mainly concentrated in the brown and turquoise modules (Figure 2d, e). Further analysis revealed four core genes in the brown module: MD06G1104100, MD10G1166300, MD05G1211000 and MD16G1178000, and three core genes in the turquoise module: MD07G1262500, MD12G1107400 and MD15G1123200 (Figure 2f). Based on these data, combined with the list of DEGs annotated as ion transporters, transcription factors, plant hormone signalling and protein kinase (Figure 2g), we selected MdWRKY53 (MD06G1104100), a member of the WRKY transcription factor family, as a key gene that functions in plant responses to KCl and MT treatment for further study.

### MdWRKY53 enhances the tolerance of transgenic apple plants to KCl stress by regulating K^+^ homeostasis in the cytoplasm

**Figure 3:**
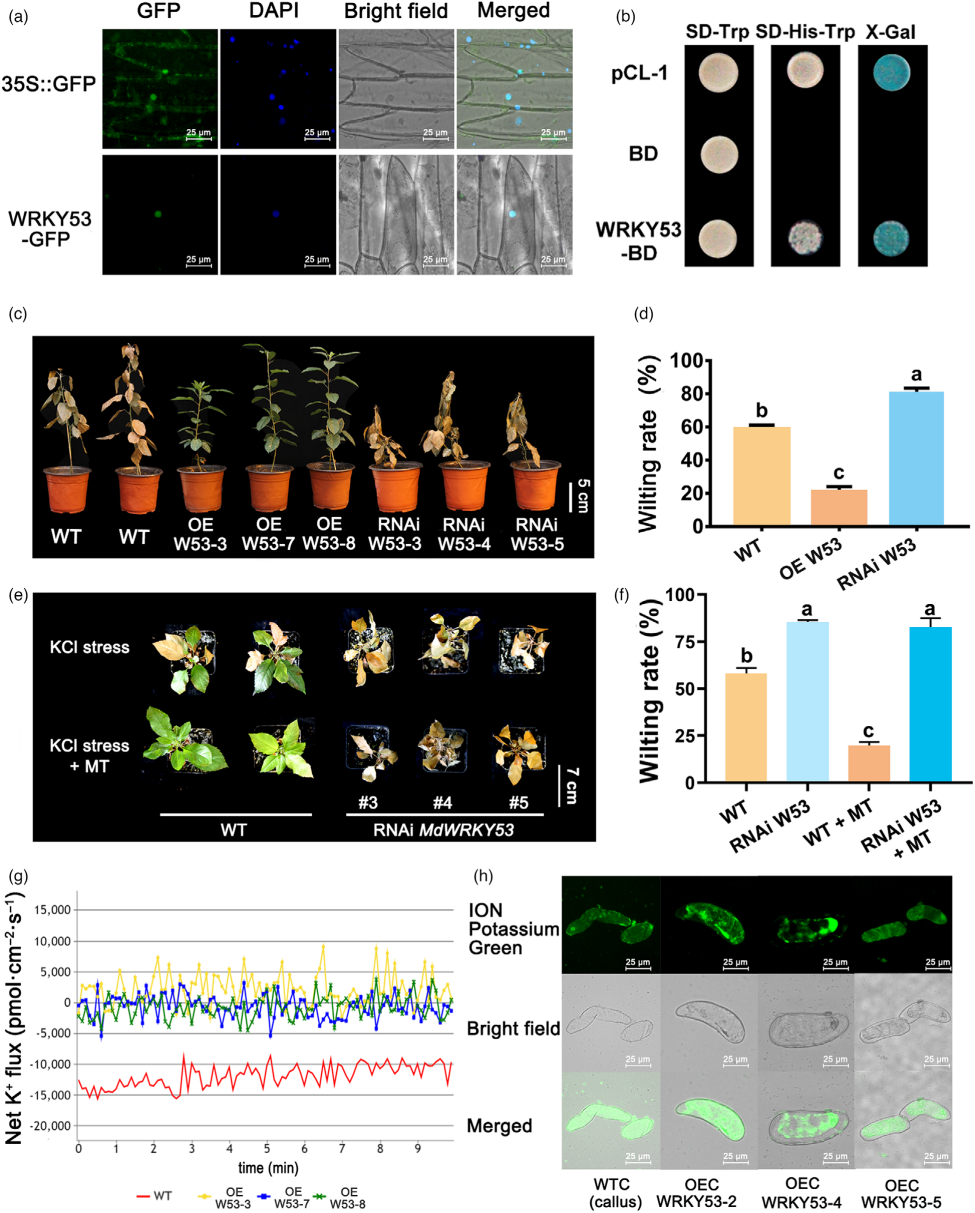
MdWRKY53 enhances KCl tolerance of transgenic apple plants by regulating K^+^ homeostasis in cytoplasm. (a) Subcellular localization of MdWRKY53‐GFP. (b) Transcriptional activity of MdWRKY53‐BD. Phenotype (c) and wilting rate (d) of OE and RNAi *MdWRKY53* transgenic lines and wild‐type plants under KCl stress. Phenotype (e) and wilting rate (f) of RNAi *MdWRKY53* transgenic lines and wild‐type ‘GL‐3’ plants under KCl stress and exogenous MT treatment. (g) Net K^+^ flux of OE *MdWRKY53* transgenic lines and wild‐type plants under KCl stress. (h) K^+^ distribution of OE *MdWRKY53* transgenic and WTC under KCl stress. Data represent the means ± SD of triplicate experiments. Different lowercase letters indicate significant differences according to Tukey's HSD (*P* < 0.05).

We performed reverse transcription quantitative PCR (RT–qPCR) to examine the expression of *MdWRKY53* in *Malus hupehensis* seedlings under KCl stress and exogenous MT treatment. *MdWRKY53* expression was significantly induced under KCl stress in both leaves and roots. Upon the application of exogenous MT, *MdWRKY53* expression continued to be up‐regulated in both the leaves and roots (Figure S4c). In addition, we also determined the expression level of *MdWRKY53* in *Qingzhen 1*, *Malus hupehensis* and *M26*. The results found that the expression level of *MdWRKY53* was positively correlated with KCl resistance, which was highest in *Qingzhen 1* and lowest in M26. Moreover, exogenous MT also significantly induced the expression of *MdWRKY53* under KCl stress in *Qingzhen 1*, *Malus hupehensis* and *M26* compared with no MT treatment (Figure S4f). We then fused MdWRKY53 to green fluorescence protein and observed that the MdWRKY53‐GFP fusion protein was localized in nucleus (Figure 3a). A transactivation assay indicated that MdWRKY53 has transcriptional activation activity in yeast (Figure 3b). These results indicated that MdWRKY53 is a transcription activator that localizes to the nucleus.

To investigate the role of MdWRKY53 in apple under KCl stress, we generated transgenic GL3 apple lines overexpressing *MdWRKY53* or expressing an *MdWRKY53*‐RNAi construct. *MdWRKY53* expression was significantly higher in three *MdWRKY53* overexpression lines (OE W53‐3, OE W53‐7, OE W53‐8) and significantly lower in three RNAi lines (RNAi W53‐3, RNAi W53‐4, RNAi W53‐5) than in the control (Figure S5a). We subjected the six transgenic apple lines and control apple plants to 50 mm KCl stress for 60 days and examined their responses. The three OE‐W53 apple lines kept growing and their leaves remained green, while the control plants were substantially wilted. The wilting of the RNAi‐W53 apple lines was more severe compared to the control, and their leaves could not spread out (Figure 3c). The wilting rates were consistent with these phenotypes. Indeed, the wilting rate of OE‐W53 plants was as low as 20.7%, which was significantly lower than that of WT (58.5%) and RNAi‐W53 plants (80.5%) (Figure 3d). We also applied exogenous MT to RNAi‐W53 and wild‐type apple plants under KCl stress, and then tested their recovery. Exogenous MT diminished the extent of wilting in wild‐type apple plants but had no substantial effects in RNAi‐W53 plants (Figure 3e). The wilting rate measurements supported these results: the wilting rate of wild‐type plants decreased from 57.7% to 20.1% after MT treatment. However, we observed no significant difference in wilting rate between RNAi‐W53 plants before and after MT treatment (Figure 3f). These results further confirm the key role of MdWRKY53 in the MT signalling pathway.

We then measured net K^+^ flux in the root tips of transgenic OE‐W53 and control apple plants under KCl stress. The K^+^ flux of the wild type was −12275.6 pmol cm^*−*2^ s^*−*1^ within 10 min of treatment, while that of OE W53‐3, OE W53‐7, OE W53‐8 transgenic apple lines was 1472.7, −716.1 and −378.0 pmol cm^−2^ s^−1^, respectively (Figure 3g). These results indicate that more K^+^ efflux occurred in OE‐W53 transgenic plants under KCl stress compared to the wild type.

To investigate the role of MdWRKY53 in K^+^ distribution, we generated three apple callus lines (OEC W53‐2, OEC W53‐4, OEC W53‐5) with higher *MdWRKY53* expression than the control (Figure S5b). As shown in Figure 3h, K^+^ ions in WT apple callus are mainly distributed in the cytoplasm after 6 h of 50 mm KCl treatment. By contrast, K^+^ in the *MdWRKY53* overexpression callus lines was substantially concentrated in the vacuoles. These results suggest that MdWRKY53 promotes the compartmentalization of K^+^ into vacuoles under KCl stress.

*AtWRKY41* is a homologous gene of *MdWRKY53* in *Arabidopsis*, and we also explore the function of *AtWRKY41* under KCl stress. The OE *AtWRKY41* and *wrky41* knock out mutants were obtained (Figure S5c), and the phenotype under 50 mm KCl showed that *AtWRKY41* played positive role in *Arabidopsis* response to KCl stress (Figure S5d). The phenotype was also supported by wilting rate detection (Figure S5e). Furthermore, we also applied exogenous MT to *wrky41* and Col‐0 under KCl stress. The results showed that application of exogenous MT significantly decreased the wilting rate of Col‐0 under KCl stress. However, the wilting rate and fresh weight of *wrky41* seedlings had no significant change with/without MT treatment (Figure S5f‐h). These results further suggested that WRKY53‐mediated MT‐promoted plant against KCl stress in different species might be conserved.

### Identification and validation of target genes downstream of MdWRKY53

**Figure 4:**
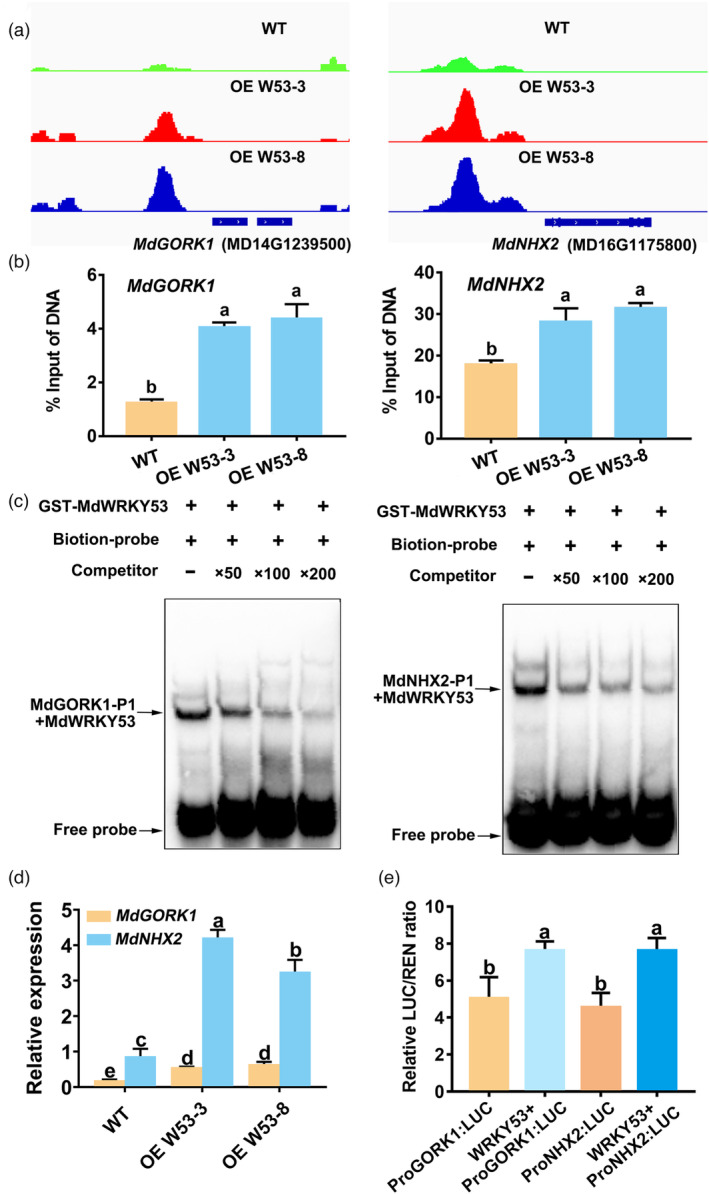
Screening and validation of MdWRKY53 downstream target genes *MdGORK1* and *MdNHX2*. (a) Two downstream genes *MdGORK1* and *MdNHX2* of MdWRKY53 were screened out by chromatin immunoprecipitation followed by sequencing (ChIP‐seq). Green represents the peak diagram of WT, while red represents the peak diagram of OE W53‐3 transgenic line, and blue represents the peak diagram of OE W53‐8 transgenic line. (b) ChIP–qPCR assay to verify the binding of MdWRKY53 to the promoters of *MdGORK1* and *MdNHX2 in vivo*, respectively. (c) Electrophoretic mobility shift assay (EMSA) to verify the binding of MdWRKY53 to the promoters of *MdGORK1* and *MdNHX2 in vitro*, respectively. The upper arrow indicates the position of a protein/DNA complex after the incubation of a biotin‐labelled DNA probe with GST‐MdWRKY53 protein. The lower arrow indicates the unbound free probe. (d) Relative expression of *MdGORK1* and *MdNHX2* in transgenic apple plants overexpressing *MdWRKY53* and wild‐type plants. (e) Relative LUC activity in transiently transformed *Nicotiana benthamiana* leaves overexpressing *Pro MdGORK1*, *Pro MdGORK1 + MdWRKY53*, *Pro MdNHX2* and *Pro MdNHX2 + MdWRKY53* fused to pGreenII 0800‐LUC vector, respectively. Data represent the means ± SD of triplicate experiments. Different lowercase letters indicate significant differences according to Tukey's HSD (*P* < 0.05).

To explore the downstream mechanism of MdWRKY53‐regulated K^+^ homeostasis, we performed ChIP‐seq experiments using OE W53‐3, OE W53‐8, and wild‐type apple plants with anti‐MdWRKY53 polyclonal antibodies. From the analysis of this data, we identified two key downstream target genes, *MdGORK1* and *MdNHX2*. We observed the enrichment of MdWRKY53 at the −1086 to −816 bp region in the *MdGORK1* promoter and the −754 to −484 bp region in the *MdNHX2* promoter (Figure 4a). Similar to *MdWRKY53*, the expression of *MdGORK1* and *MdNHX2* were also up‐regulated by KCl and MT treatment (Figure S4d,e). Moreover, their expression levels were also positively correlated with KCl resistance in *Qingzhen 1*, *Malus hupehensis* and M26 (Figure S4g, h). Subsequently, we used ChIP–qPCR and electrophoretic mobility shift assays (EMSAs) to validate the direct binding of MdWRKY53 to the *MdGORK1* and *MdNHX2* promoters *in vivo* and *in vitro*. We confirmed the enrichment of MdWRKY53 at the *MdGORK1* and *MdNHX2* promoters in OE W53‐3 and OE W53‐8 compared with the wild‐type apple plants (Figure 4b). In the EMSA, recombinant WRKY53‐GST (glutathione S‐transferase) is bound to the *MdGORK1* and *MdNHX2* promoter probes *in vitro*. The addition of unlabeled competitor probes reduced the extent of this binding (Figure 4c). These results suggest that MdWRKY53 binds to the *MdGORK1* and *MdNHX2* promoters *in vivo* and *in vitro*.

As MdWRKY53 is a transcriptional activator, we performed RT–qPCR and LUC activity assays to investigate the effect of MdWRKY53 in regulating *MdGORK1* and *MdNHX2* expression. The expression levels of *MdGORK1* and *MdNHX2* were significantly higher in the OE W53 transgenic lines compared to wild‐type plants (Figure 4d). We also infiltrated Agrobacterium cultures harbouring the pairs of vectors *ProMdGORK1*:*LUC* + *MdWRKY53*‐SK and *ProMdNHX2*:*LUC* + *MdWRKY53*‐SK into *Nicotiana benthamiana* leaves for LUC activity assays; *ProMdGORK1*:*LUC* + SK and *ProMdNHX2*:*LUC* + SK were used as controls. We measured significantly higher relative LUC activity in leaves co‐infiltrated with *ProMdGORK1*:*LUC* + *MdWRKY53*‐SK or *ProMdNHX2*:*LUC* + *MdWRKY53*‐SK compared with the combinations *ProMdGORK1*:*LUC* + SK or *ProMdNHX2*:*LUC* + SK (Figure 4e), confirming the positive role of MdWRKY53 in regulating *MdGORK1* and *MdNHX2* transcription. Taken together, these results suggest that MdWRKY53 directly binds to the *MdGORK1* and *MdNHX2* promoters and enhances the transcription of these key downstream target genes.

To further confirm the relationship between MdWRKY53 and *MdGORK1/MdNHX2*. We obtained the stable transgenic apple callus OEC *MdWRKY53* + RNAi *MdGORK1* and OEC *MdWRKY53* + RNAi *MdNHX2* (Figure S6b). The phenotype of transgenic OEC *MdWRKY53*, OEC *MdWRKY53* + RNAi *MdGORK1*, OEC *MdWRKY53* + RNAi *MdNHX2*, and wild‐type apple callus (WTC) under KCl stress showed that the KCl resistance of OEC *MdWRKY53* + RNAi *MdGORK1* and OEC *MdWRKY53* + RNAi *MdNHX2* were significantly lower than OEC *MdWRKY53* (Figure S6a), which was also supported by browning rate detection (Figure S6c). These results further confirmed the regulatory relationship of *MdGORK1/MdNHX2* by MdWRKY53.

### MdGORK1 improves KCl tolerance in apple plants by enhancing K^+^ efflux

**Figure 5:**
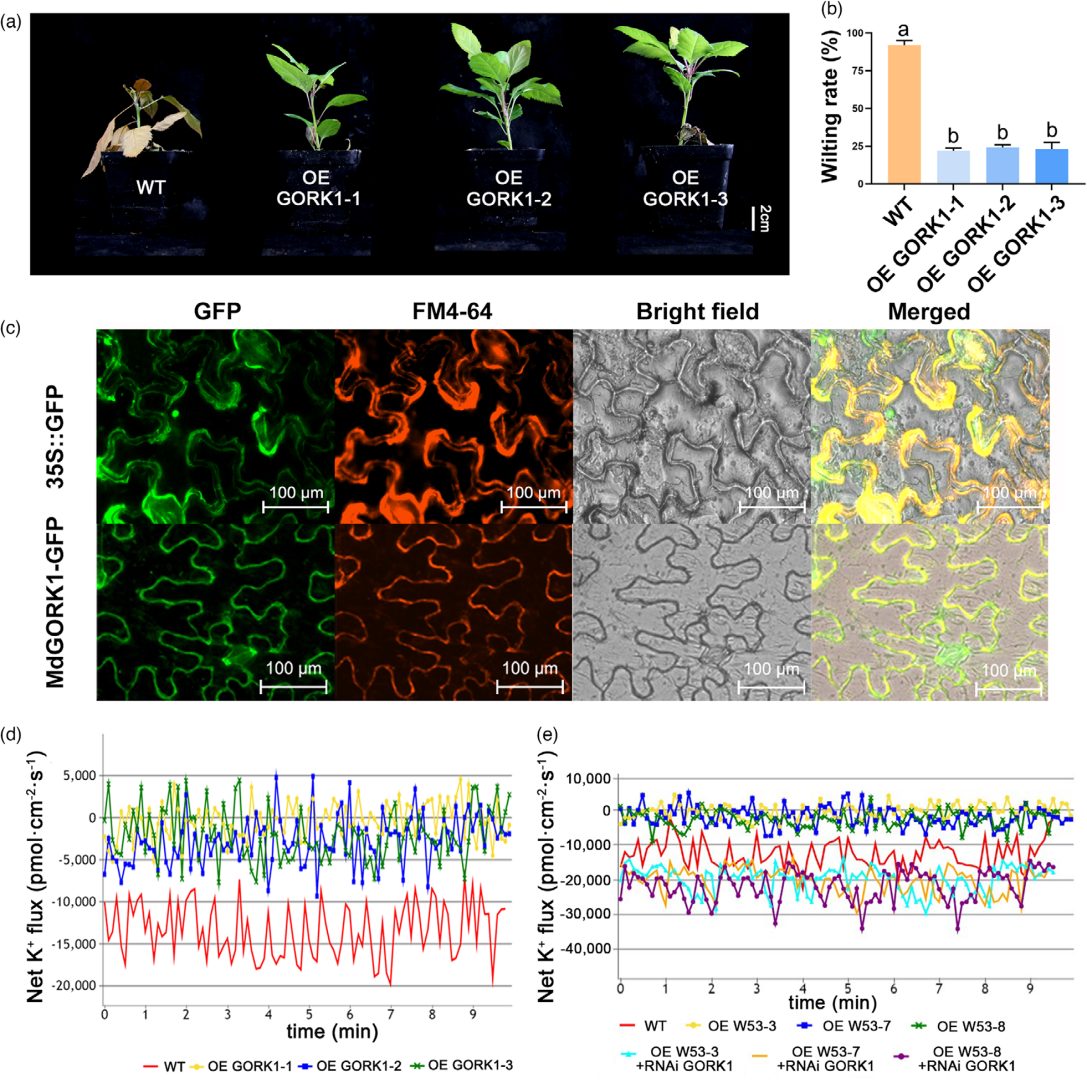
*MdGORK1* improves KCl tolerance of apple plants through enchancing K^+^ efflux. Phenotype (a) and wilting rate (b) of OE *MdGORK1* transgenic lines and wild‐type plants under KCl stress. (c) Subcellular localization of MdGROK1‐GFP. (d) Net K^+^ flux of OE *MdGORK1* transgenic lines and wild‐type plants under KCl stress. (e) Net K^+^ flux of OE *MdWRKY53*, OE *MdWRKY53* + RNAi *MdGORK1* transgenic apple roots under KCl stress. Data represent the means ± SD of triplicate experiments. Different lowercase letters indicate significant differences according to Tukey's HSD (*P* < 0.05).

We generated three transgenic apple lines overexpressing *MdGORK1* (OE *GORK1*‐1, OE *GORK1*‐2, OE *GORK1*‐3), and confirmed that they have higher *MdGORK1* expression levels compared with wild‐type apple plants (Figure S6d). We treated the three transgenic lines and control plants with 50 mm KCl stress for 30 days. As shown in Figure 5a, the three transgenic lines showed substantially better growth compared to the wild type under KCl stress. Analysis of wilting rates supported these phenotypes (Figure 5b). These results indicate that overexpressing *MdGORK1* improves the KCl tolerance of transgenic apple plants.

We then examined the subcellular localization of MdGORK1‐GFP in *Nicotiana benthamiana* leaves. As shown in Figure 5c, MdGORK1‐GFP signals overlapped with the plasma membrane marker FM4‐46, indicating that MdGORK1 localizes to the plasma membrane. We also measured K^+^ flux in transgenic apple plants overexpressing *MdGORK1*. The K^+^ flux of lines OE *GORK1*‐1, OE *GORK1*‐2, OE *GORK1*‐3 was −711.9, −3316.1, and −1978.0 pmol cm^−2^ s^−1^, respectively, which were significantly higher than the value in wild‐type apple plants (−13219.3 pmol cm^−2^ s^−1^) (Figure 5d). These data suggest that MdGORK1 localizes to the plasma membrane and enhances K^+^ efflux to increase the tolerance of apple plants to KCl stress. Furthermore, we transient transformed RNAi *MdGORK1* in OE *MdWRKY53* apple roots and detected the net K^+^ flux. The results showed that the net K^+^ flux in OE *MdWRKY53* + RNAi *MdGORK1* apple roots was substantially lower than that in OE *MdWRKY53* apple roots (Figure 5e). These results suggested that MdWRKY53 regulated K^+^ efflux by regulating the expression of *MdGORK1*.

### MdNHX2 improves KCl tolerance in apple plants by compartmentalizing K^+^

**Figure 6:**
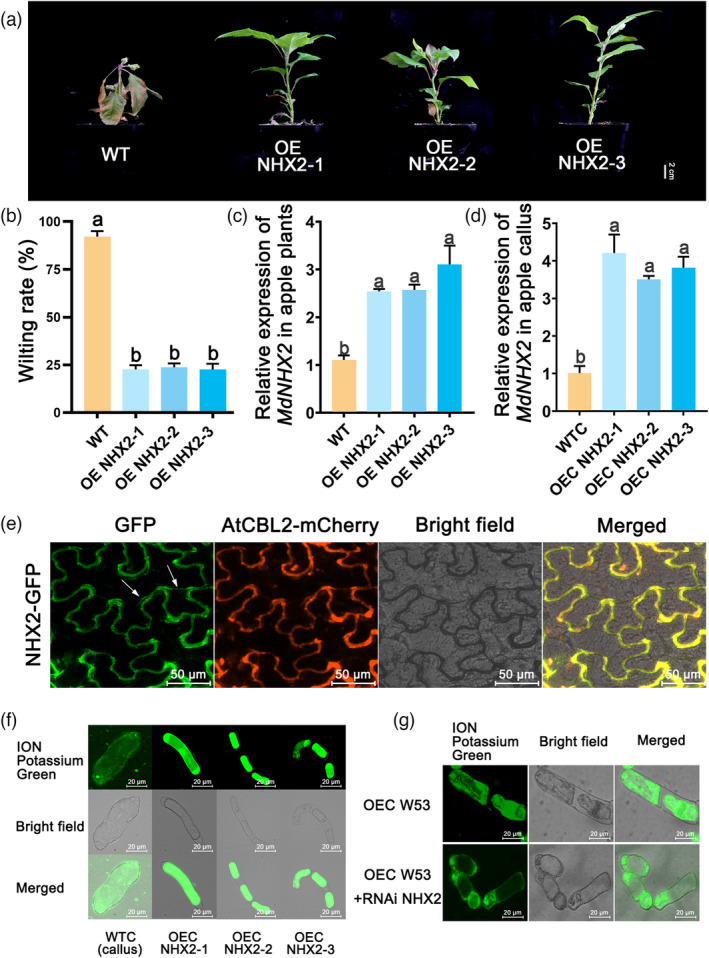
*MdNHX2* improves KCl tolerance of apple plants through K^+^ compartmentalization. Phenotype (a) and wilting rate (b) of OE *MdNHX2* transgenic lines and wild‐type plants under KCl stress. (c) Relative expression of *MdNHX2* in transgenic apple plants overexpressing *MdNHX2* and wild‐type plants. (d) Relative expression of *MdNHX2* in transgenic apple callus overexpressing *MdNHX2* and WTC. (e) Subcellular localization of MdNHX2‐GFP. AtCBL2‐mCherry was a tonoplast marker. (f) K^+^ distribution of OE *MdNHX2* transgenic callus (OEC *NHX2*) and wild‐type apple callus (WTC) under KCl stress. (g) K^+^ distribution of OE *MdWRKY53* (OEC *W53*) and OE *MdWRKY53* + RNAi *MdNHX2* transgenic apple callus (OEC *W53* + RNAi *NHX2*) under KCl stress. Data represent the means ± SD of triplicate experiments. Different lowercase letters indicate significant differences according to Tukey's HSD (*P* < 0.05).

To explore the role of MdNHX2 in apple under KCl stress, we generated three transgenic apple lines overexpressing *MdNHX2* (OE *NHX2*‐1, OE *NHX2*‐2 and OE *NHX2*‐3) and three transgenic apple callus lines overexpressing *MdNHX2* (OEC *NHX2*‐1, OEC *NHX2*‐2, and OEC *NHX2*‐3), with higher *MdNHX2* expression levels than the control (Figure 6c, d). After 30 days of KCl stress treatment, the transgenic plants overexpressing *MdNHX2* showed much better growth than wild‐type apple plants (Figure 6a). This phenotype was consistent with the wilting rates of these plants (Figure 6b). MdNHX2–GFP was found localized in the tonoplast (Figure 6e). We observed no significant difference in K^+^ flux in the rhizosphere between transgenic apple plants overexpressing *MdNHX2* and wild‐type plants, indicating that overexpressing *MdNHX2* has no effect on K^+^ influx into or efflux out of cells (Figure S6e). Finally, to further investigate the role of MdNHX2 in K^+^ transport, we stained the transgenic apple callus under KCl stress with ION K Green. Although K^+^ was mainly distributed in the cytoplasm in wild‐type callus, most K^+^ was compartmentalized into the vacuole in transgenic apple callus overexpressing *MdNHX2*, which was similar to the pattern in *MdWRKY53‐*overexpressing lines (Figure 6f). These results indicate that MdNHX2 enhances the tolerance of transgenic apple plants to KCl stress by compartmentalizing K^+^ into the vacuole. Furthermore, we also detected the K^+^ distribution of OEC *MdWRKY53* and OEC *MdWRKY53* + RNAi *MdNHX2* transgenic apple callus under KCl stress. It was found that K^+^ was mostly compartmentalized into vacuole in OEC *MdWRKY53* transgenic apple callus, while K^+^ was widely distributed in cytoplasm in OEC *MdWRKY53* + RNAi *MdNHX2* transgenic apple callus (Figure 6g). These results further suggested that MdWRKY53 promoted vacuole compartmentalization of K^+^ by regulating *MdNHX2* expression.

## Discussion

The application of plant growth regulators is an effective approach for improving abiotic stress tolerance in crops, offering a rapid reduction of the inhibitory effects of these stressors that does not rely on genetic engineering (Rademacher, 2015). MT, which is related to potent naturally occurring ROS and reactive nitrogen species scavengers in plants, improves plant tolerance against a variety of abiotic stresses (Zheng *et al*., 2021b). However, little is known about the role of MT in KCl stress. In the present study, we discovered that the application of MT substantially alleviated KCl stress in high‐resistance apple rootstock *Qingzhen 1*, medium‐resistance rootstock *Malus hupehensis* and KCl‐sensitive rootstock M26 (Figures 1 and S1), indicating its effective effect in apple under KCl stress. In addition, treatment with 100 μm MT produced the best effect, with the lowest wilting rate and the highest fresh weight, dry weight and root length compared to 10 or 200 μm MT (Figure 1). These results highlight the dose‐dependent effects of MT on KCl tolerance in apple seedlings. It is indeed important to use the appropriate concentrations of plant growth regulators, as they can be ineffective or have inhibitory or even detrimental effects when applied at improper concentrations (Li *et al*., 2021a). For instance, root growth in *Brassica juncea* was stimulated in response to low concentrations of MT (0.1 mm), whereas a high concentration (100 mm) inhibited root growth (Chen *et al*., 2009). Brassinosteroid‐facilitated xylem development also occurs in a dosage‐dependent manner within a certain concentration range in the pine species *Pinus massoniana* seedlings (Fan *et al*., 2021). Therefore, our study provides a novel means and concentration of melatonin for alleviating KCl stress in apple seedlings.

KCl stress can induce ion toxicity and osmotic stress in plants, leading to ROS accumulation and oxidative stress (Yang and Guo, 2018). For ion toxicity, our results indicate that the contents of K, Na, Ca and Mg significantly increased in *Malus hupehensis* seedlings under KCl stress. The K content increased nearly three‐fold, inducing serious K toxicity. When treated with exogenous MT, the K content of *Malus hupehensis* seedlings decreased by 22%, pointing to the important role of MT in K^+^ homeostasis (Figure S3a). Indeed, previous studies indicated that MT functions in ion homeostasis in plants. MT‐mitigated cadmium phytotoxicity by increasing cadmium levels in the cell wall of tomato (*Solanum lycopersicum*) plants (Hasan *et al*., 2015). In wheat, MT ameliorates aluminium toxicity by decreasing aluminium contents in root tips (Sun *et al*., 2020). Moreover, MT antagonizes abscisic acid activity to promote seed germination by regulating Ca^2+^ efflux (Li *et al*., 2021b). However, little is known about the role of MT in K^+^ homeostasis. Our results found that K^+^ flux and vacuole compartmentalization of K^+^ were key KCl‐response mechanism in apple, due to that the K^+^ flux and K^+^ compartmentalization were positively correlated with KCl resistance of apple rootstocks (Figure S1h, i). Therefore, we explored K^+^ flux and distribution in three different KCl resistance apple seedlings under KCl stress and exogenous MT treatment. MT significantly enhanced the efflux of K^+^ out of cells and compartmentalized K^+^ into the vacuole to alter the distribution of K^+^ under KCl stress (Figures 1 and S1), representing a novel mechanism for the role of MT in K^+^ homeostasis.

The roles of MT in plant stress responses often depend on its signal transduction pathways (Sun *et al*., 2021). MT‐related receptors, kinases and transcription factors have been reported in many plant species. Nevertheless, the molecular mechanism of MT in KCl stress remains unclear. Here, we identified the transcription factor gene *MdWRKY53*, which was responsive to KCl stress and MT treatment, by WGCNA (Figure 2). We used this gene to transform apple plants and found that it positively regulated the response to KCl stress. The wilting of RNAi‐*W53* transgenic apple plants could not be recovered by exogenous MT treatment, further indicating that *MdWRKY53* is the core gene in the MT signal transduction pathway under KCl stress (Figure 3). *AtWRKY41*, as the *Arabidopsis* homologous gene of *MdWRKY53*, also played positive role in *Arabidopsis* response to KCl stress. Moreover, MT had no effect on the wilting phenotype of *wrky41* under KCl stress (Figure S5), further suggesting that WRKY53‐mediated MT‐promoted plant against KCl stress in different species might be conserved. In addition, in a K^+^ flux experiment, we observed more K^+^ efflux under KCl stress in OE‐*W53* transgenic plants compared to the wild type. Moreover, K^+^ in *MdWRKY53*‐overexpressing callus lines concentrated in the vacuole, while it was concentrated in the cytoplasm of WTC (Figure 3). The function of MdWRKY53 in maintaining the balance of K^+^ is consistent with the role of MT in K^+^ homeostasis, indicating that MdWRKY53 plays an important role in MT‐regulated K^+^ homeostasis under KCl stress. Consistent results were obtained for AtWRKY41 in Arabidopsis. A few studies have examined the relationship between WRKY transcription factors and MT. Wei *et al*. (2018b) found that MT biosynthesis enzymes recruit WRKY transcription factors to regulate MT accumulation and transcriptional activity on W‐box in cassava, indicating WRKY involved in MT biosynthesis. In addition, *EjWRKY17* was significantly up‐regulated in leaves upon MT treatment during drought stress in loquat (*Eriobotrya japonica*) (Wang *et al*., 2021a), suggesting that this WRKY transcription factor takes part in MT signal transduction. Here, we demonstrated that MdWRKY53 plays a key role in MT regulating K^+^ homeostasis to response KCl stress in apple.

In signal transduction pathways, transcription factors receive upstream signals and regulate the transcription of downstream target genes, taking part in plant development and stress responses (Romani and Moreno, 2021). The downstream genes of various WRKY transcription factors under salt stress have been reported, mainly focusing on NaCl stress. In Arabidopsis, WRKY transcription factors improve NaCl tolerance by regulating the expression of *AtKUP2* (Rajappa *et al*., 2020). SbWRKY50 directly binds to the promoter of *SbSOS1* (*Salt overly sensitive 1*) and participates in the salt‐stress response of Sorghum (*Sorghum bicolor*) (Song *et al*., 2020). In tomato, *SlWRKY3* is rapidly induced by NaCl and KCl treatments to regulate the K^+^:Na^+^ ratio (Hichri *et al*., 2017), preliminary indicating WRKY was involved in plant response to KCl stress. However, the downstream target genes of WRKY transcription factors under KCl stress were unknown until now. In the present study, we determined that MdWRKY53 is a nucleus‐localized transcriptional activator that plays an important role in K^+^ homeostasis (Figure 3). We identified two genes (*MdGORK1* and *MdNHX2*) downstream of MdWRKY53, which were closely related to K^+^ homeostasis, by ChIP‐seq. We then performed ChIP–qPCR and EMSA to confirm the direct binding of MdWRKY53 to the promoters of *MdGORK1* and *MdNHX2 in vivo* and *in vitro* (Figure 4). Furthermore, our genetic experiments further confirmed the transcriptional regulation of *MdGORK1* and *MdNHX2* by MdWRKY53 under KCl stress (Figure S6), and MdWRKY53 enhanced the K^+^ efflux and vacuole compartmentalization of K^+^ mainly through regulating the function of *MdGORK1* and *MdNHX2*, respectively (Figures 5 and 6). Therefore, we identified two novel downstream target genes of a WRKY transcription factor under KCl stress. One of these target genes is *MdGORK1*, encoding a member of the Shaker family of K channels; these K channels are involved in stress responses and the regulation of stomatal movements (Hosy *et al*., 2003; Zhao *et al*., 2019). A previous study indicated that GORK was sensitive to the external KCl concentration (Eisenach *et al*., 2014). In the current study, RT–qPCR indicated that *MdGORK1* was up‐regulated by MT and KCl treatment (Figure S4), and overexpressing *MdGORK1* significantly enhanced KCl tolerance in apple plants compared with the wild type (Figure 5), further confirming that MdGORK1 is involved in KCl responses. To further investigate the underlying mechanism, we examined the localization of MdGORK1. This protein localized to the plasma membrane and enhanced K^+^ efflux under KCl stress, as revealed by detecting ion flow in roots (Figure 5). Lim *et al*. (2019) reported that GORK is targeted to the plasma membrane, where it regulates K^+^ efflux, leading to stomatal closure in Arabidopsis. We identified a similar function for a GORK protein in the woody plant apple under KCl stress.

Another downstream gene encodes the Na^+^ (K^+^)/H^+^ antiporter MdNHX2; Na^+^ (K^+^)/H antiporters are critical for exchanging Na^+^ or K^+^ between the cytoplasm and vacuole (Leidi *et al*., 2010). Previous studies have reported that NHX family members mainly function in Na^+^/H^+^ antiport in plants in response to NaCl stress. However, new opinion considered that Arabidopsis AtNHX7 and AtNHX8 localize to the plasma membrane and are responsible for Na^+^/H^+^ transport, while AtNHX1‐4 are located at the tonoplast and control vacuolar pH and K^+^ uptake to regulate stomatal function, flower development and reproduction (Barragán *et al*., 2012; Bassil *et al*., 2011; Reguera *et al*., 2013). Few studies have focused on the roles of NHX proteins in plants under KCl stress. Here, we showed that *MdNHX2* was induced by KCl and MT treatment, and overexpressing *MdNHX2* increased KCl tolerance, indicating it plays a positive role in apple under KCl stress (Figures 6 and S4). MdNHX2 localized to the tonoplast, and most K^+^ was compartmentalized in the vacuoles in transgenic apple callus overexpressing *MdNHX2* under KCl stress, whereas K^+^ was mainly distributed in the cytoplasm in WTC (Figure 6). These findings provide important new evidence for the role of NHX2 in K^+^ transport between the cytoplasm and vacuole in apple plants. Taken together, we identified two downstream genes of the WRKY transcription factor MdWRKY53 and showed that MdWRKY53 directly bound to the promoters of *MdGORK1* and *MdNHX2* to enhance their transcription, resulting in increased K^+^ efflux and the compartmentalization of K^+^ in the vacuole to enhance KCl tolerance.

**Figure 7:**
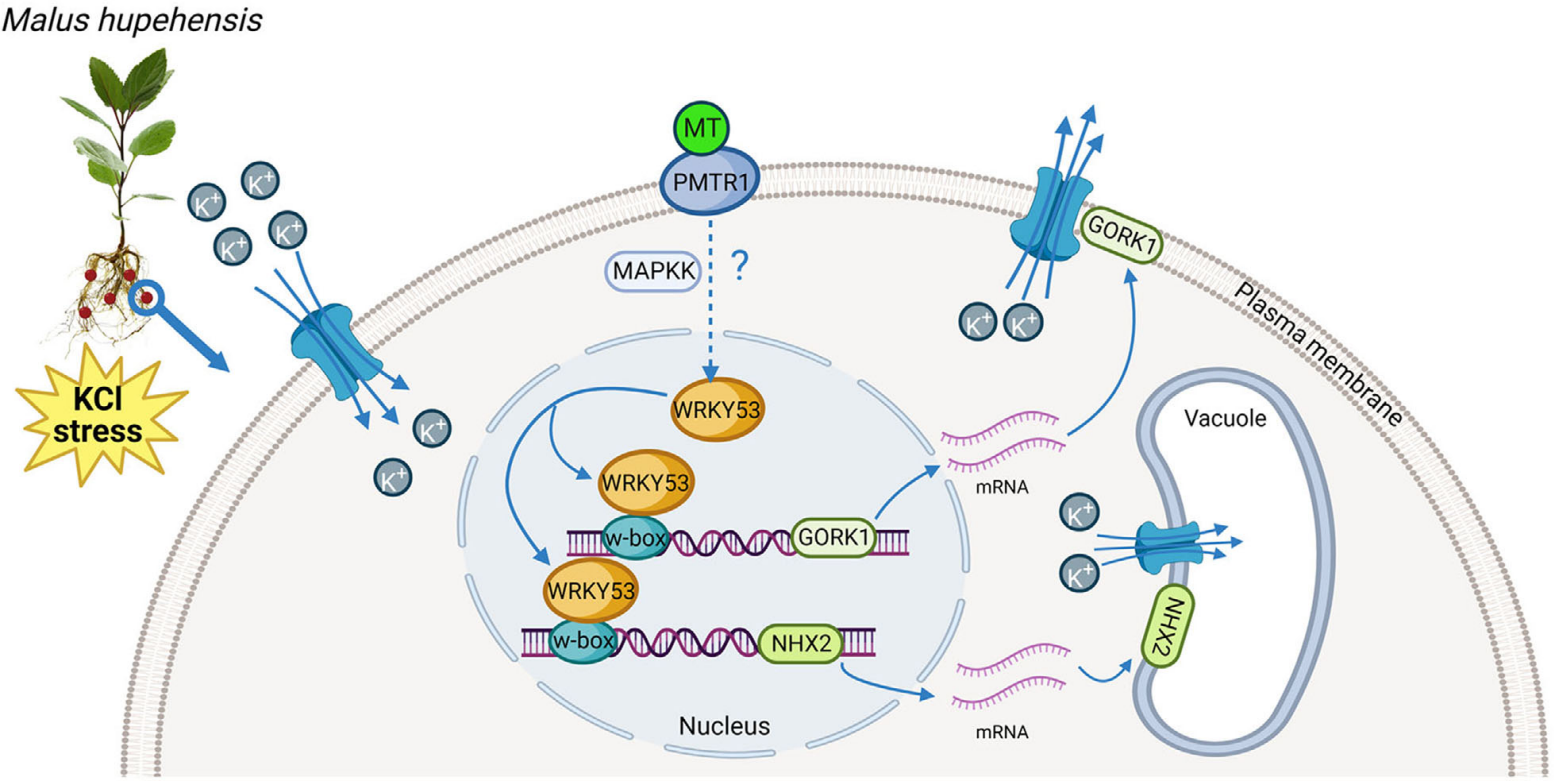
Proposed MT‐WRKY53‐GORK1/NHX2‐K^+^ module regulates K^+^ homeostasis to enhance KCl stress tolerance in apple. MT binds to the receptor PMTR1, and may transmit the signal to the key transcription factor MdWRKY53, which located in nucleus, through MAPK cascade phosphorylation. On one hand, MdWRKY53 can directly bind to the promoters of *MdGORK1* and increase its transcript to increase K^+^ efflux out of cells. On the other hand, MdWRKY53 can also directly bind to the promoters of *MdNHX2* and enhance its transcript to compartmentalize K^+^ into vacuole. Further keeping the K^+^ homeostasis in cytoplasm resulting in enhanced KCl tolerance of apple plants.

In conclusion, we identified the novel MT‐WRKY53‐GORK1/NHX2‐K^+^ homeostasis regulatory module, which functions in KCl stress in apple. In response to exogenous MT treatment in plants under KCl stress, MT transmits the stress signal to the core nucleus‐localized transcription factor MdWRKY53. MdWRKY53 directly binds to the promoter of *MdGORK1* and enhances its transcription; the plasma membrane‐localized MdGORK1 protein increases K^+^ efflux out of cells. MdWRKY53 also directly binds to the promoter of *MdNHX2* and increases its expression; the tonoplast‐localized MdNHX2 protein compartmentalizes K^+^ into vacuoles. These processes help maintain K^+^ homeostasis in the cytoplasm, resulting in enhanced KCl tolerance in apple plants (Figure 7). However, the signal that is transmitted from MT to MdWRKY53 under KCl stress is still unclear. RNA‐seq showed that the expression of *MdMAPKK1* significantly changed under KCl and exogenous MT treatment (Figure 2), pointing to its potential role in MT signal transduction. Several MAPKKK‐MKK‐MPK and MPK‐WRKY modules and their functions have been well studied. For instance, Arabidopsis MPK3 and MPK6 play major roles in fungus‐induced camalexin accumulation by phosphorylating WRKY33 (Mao *et al*., 2011). Therefore, further studies will focus on the upstream signalling pathway of MT to WRKY in plants under abiotic stress.

## Materials and methods

### Plant materials and growth conditions

*Malus hupehensis* seeds with a 96% apomixis rate were sown on nutrient soil after vernalization for 2 months. When the seedlings had developed four leaves, they were transferred to pots and irrigated with Hoagland nutrient solution every third day. Two weeks after transplanting, the seedlings were selected for KCl stress and exogenous MT treatment.

‘Gala3’ (GL3) (*Malus domestica*), *Malus baccata*, *Malus xiaojinensis*, M9, *Malus zumi*, M26, *Qingzhen 1* and *Malus hupehensis* apple plants were cultured on Murashige and Skoog (MS) medium containing 0.5 mg/L indole‐3‐butyric acid (IBA) and 0.5 mg/L 6‐benzylaminopurine (6‐BA) and sub‐cultured every three weeks. The plants were rooted and transplanted into soil as described by Zheng *et al*. (2018). The growth conditions of all plants were set to 23 °C, 55% relative humidity, with a light intensity of 100 μmol m^−2^ s^−1^.

Apple calli were grown on MS medium containing 0.4 mg/L 6‐BA and 1.5 mg/L 2,4‐dichlorophenoxyacetic acid (2,4‐D). The subculture and growth conditions were described by Zheng *et al*. (2018).

### Exogenous MT and KCl stress treatments

A total of 250 *Malus hupehensis* seedlings with similar growth status were evenly divided into five groups. Group I was irrigated with Hoagland nutrient solution and served as the control. Group II was irrigated with Hoagland nutrient solution +50 mm KCl. Groups III–V were subjected to the same treatment as Group II but with the addition of exogenous MT (SolarBio, Beijing, China) at concentrations of 10, 100, or 200 μm, respectively. MT was dissolved in ethanol at a concentration of 10 mm and stored at −20 °C. The plants were sprayed and irrigated with MT every 3 days. After 30 days of treatment, the phenotype and physiological data of wilting rate, fresh weight, dry weight, and root length were recorded. Determination of wilting rate as described by Li *et al*. (2021c). Plant wilting rate is the percentage of the number of wilting or dead plants to the total seedling population.

For the KCl stress of seven kinds of apple rootstocks. 30 tissue culture seedlings of every kind of apple rootstock after being transplanted into the soil for two weeks were used for KCl stress. They were treated with 50 mm KCl for 20 days. Then the phenotype, wilting rate, fresh weight and dry weight were recorded. The KCl stress and MT treatment of M26, *Qingzhen 1* and *Malus hupehensis* were conducted as described above.

For KCl stress treatment of transgenic and control apple plants, after rooting, transplanting to soil, and 1 month of growth, transgenic apple plants overexpressing *MdWRKY53* or harbouring a *MdWRKY53*‐RNAi construct and control plants were subjected to 50 mm KCl stress for 60 days. In addition, transgenic apple plants overexpressing *MdNHX2* or *MdGORK1* and control plants were treated with 50 mm KCl stress for 30 days. After KCl stress treatment, the wilting rate was recorded. The KCl and MT treatment for wild type and RNAi *MdWRKY53* transgenic apple plants were conducted as described above.

For KCl stress for apple callus, the callus was put on MS medium containing 50 mm KCl for 30 days. Then, the phenotype and browning rate were recorded. For MT and KCl treatment for *Arabidopsis*, *Col‐0*, OE *AtWRKY41* and *wrky41* (Ding *et al*., 2014) were subjected to 50 mm KCl or 50 mm KCl + 100 μm MT for 30 days. The phenotype, wilting rate and fresh weight were recorded.

### ROS staining and measurement of antioxidant enzyme activity and osmotic index

ROS staining in roots was performed according to Zuo *et al*. (2014), with some modifications. The roots of apple seedlings after KCl stress and exogenous MT treatment for 30 days were immersed in CM‐H_2_DCFDA solution and placed under a vacuum for 10 min. The excess CM‐H_2_DCFDA was rinsed off with distilled water. After drying, the green fluorescence of roots was observed and photographed with a living plant fluorescence detector (Vilber Bio Imaging, Paris, France). H_2_O_2_ and O_2_—staining in leaves was conducted as described by Zheng *et al*. (2021a), and electrolyte leakage was determined as described previously (Su *et al*., 2020). In addition, the contents of malondialdehyde, soluble sugars, proline, SOD, POD and CAT activities were determined using specific detection kits (Grace Biotechnology Co., Ltd., Suzhou, China).

### Measurement of mineral element contents

Mineral elements were measured in 5 g apple seedling tissue after 30 days of KCl stress and exogenous MT treatment. The samples were dried for 30 min at 105 °C and baked continuously at 80 °C for 72 h. The dried seedlings were digested with 12 mL of HNO_3_ and HClO_4_ and diluted with deionized H_2_O to 25 mL. The concentrations of K, Na, Ca, phosphorus (P), Mg, iron (Fe), manganese (Mn), copper (Cu) and zinc (Zn) were determined by inductively coupled plasma emission spectrometry (ICP–ES) as described by Su *et al*. (2020).

### Measurement of net K^+^ flux

The real‐time regular net flux of K^+^ in apple plants was measured using non‐invasive micro‐test technology (Physiolyzer, Beijing, China) as described previously (Jing *et al*., 2021). The root tips were soaked in 10 mm KCl solution for 10 min. The sensor was placed at the root tip, and K^+^ flux was measured for 10 min. K^+^ flux data were recorded using imFluxes V2.0 software (Xuyue, Beijing, China).

### Visualization of cellular K^+^ distribution

ION Potassium Green (Molecular Probes, USA) was used to detect K^+^ distribution. K^+^ staining was performed as described by Oh *et al*. (2010). The apple plants and callus were treated with K^+^‐staining solution (MS medium +50 mm KCl + 10 μm ION Potassium Green) for 6 h in the dark at 23 °C, rinsed three times with deionized water, and observed by confocal microscopy (Olympus FV500, http://www.olympus‐global.com/). The excitation wavelength of ION K Green was 488 nm, and the emission wavelength was 500–530 nm.

### RNA‐seq analysis

RNA‐seq was performed using the roots and leaves of *Malus hupehensis* plants under control, KCl stress, and KCl + exogenous MT treatment for 6 h (Cao *et al*., 2021; Liu *et al*., 2023) with three independent biological replicates per tissue type. Total RNA was extracted from the samples and purified using an RNAprep Pure Plant Total RNA Extraction kit (Qiagen, Japan). RNA samples (1 μg RNA per sample) were sent to Biomarker Technologies Corporation (Beijing, China) for sequencing on an Illumina platform. Library preparation, clustering and sequencing, quality control, reads mapping, GO and KEGG enrichment analysis of DEGs, and Weighted gene co‐expression network analysis (WGCNA) were conducted as described by Li *et al*. (2021d). The reference genome was GDDH13v1.1 (https://www.rosaceae.org/species/malus/malus_×_domestica/genome_GDDH13_v1.1).

### Reverse transcription quantitative PCR (RT–qPCR)

Total RNA was extracted from the samples using an RNAprep Pure Plant Kit (Tiangen, Beijing, China), and first‐strand cDNA was synthesized from 2 μg of total RNA using a SPARKscript II RT Plus Kit (SparkJade, Jinan, China). The qPCR assay was conducted as described by Li *et al*. (2021d). The primers used for qPCR are listed in Table S3. Apple *Actin* (accession number: MDP0000774288) was used as the internal control.

### Subcellular localization and transcriptional activity analysis

For subcellular localization of MdWRKY53/MdGORK1/MdNHX2, these proteins were separately fused to green fluorescent protein (GFP) by cloning their coding sequences individually into the pMDC83 vector. The primers used are listed in Table S3. Plasmids containing *MdWRKY53/MdGORK1/MdNHX2*‐pMDC83 or pMDC83 were infiltrated into the epidermis of *Nicotiana benthamiana* leaf cells by Agrobacterium (*Agrobacterium tumefaciens*)‐mediated infiltration. After 72 h, green fluorescence was observed under an Olympus confocal microscope. The excitation wavelength was 488 nm for GFP and 405 nm for DAPI, and emission was 500 to 535 nm for GFP and 430 nm to 550 nm for DAPI (Laser, Ar‐Laser LASOS LGK 7872 ML05 and Coherent CUBE 405 nm; gain, 800 V). FM4‐64 was a plasma membrane marker. For the marker of tonoplast, AtCBL2‐mCherry was reported to be localized in tonoplast and used in this study (Yang *et al*., 2023). To examine the transcriptional activity of MdWRKY53, the *MdWRKY53* coding sequence without the stop codon was cloned into the pGBKT7 vector to generate the pBD*‐MdWRKY53* construct encoding MdWRKY53 as a fusion protein to the GAL4‐binding domain (BD). The primers used are listed in Table S3. Yeast (*Saccharomyces cerevisiae* AH109) cells harbouring *MdWRKY53‐BD*, pCL‐1 (positive control), or *BD* (negative control) were grown on synthetic defined (SD) medium—Trp and SD medium—Trp–His. Yeast cells that grew on SD medium—Trp–His were stained for β‐Galactosidase activity (SolarBio, Beijing, China).

### Generation of transgenic apple plants and callus

The coding sequences of *MdWRKY53* (MD06G1104100), *MdNHX2* (MD16G1175800) and *MdGORK1* (MD14G1239500) were amplified from *Malus hupehensis* using the primers shown in Table S3 and individually cloned into the pBI121 vector to generate the 35S:*MdWRKY53/MdNHX2/MdGORK1* constructs. The anti*‐MdWRKY53* sequence was also cloned into the pBI121 vector to generate the *MdWRKY53* RNAi construct. Introduction of the resulting vectors into Agrobacterium strain EHA105 and Agrobacterium‐mediated transformation of GL3 were described previously (An *et al*., 2021). *MdWRKY53‐*pBI121 and *MdNHX2‐*pBI121 were also transformed into ‘Orin’ apple callus as described by Zhang *et al*. (2021). After OE *MdWRKY53* transgenic apple callus were achieved, *MdGORK1‐super1300* (RNAi), *MdNHX2‐Super1300* (RNAi) were then transformed into OE *MdWRKY53* transgenic apple callus to gain OE *MdWRKY53* + RNAi *MdGORK1* and OE *MdWRKY53* + RNAi *MdNHX2* transgenic apple callus, respectively. Transgenic apple plants and callus were confirmed by PCR analysis. *MdWRKY53/MdNHX2/MdGORK1* expression levels in wild‐type and transgenic apple plants/callus were determined by RT–qPCR.

### ChIP‐seq and ChIP–qPCR analysis

ChIP assays using anti‐MdWRKY53 polyclonal antibodies were performed as described by Zheng *et al*. (2018). The recovered DNA was subjected to paired‐end sequencing on an Illumina TruSeq platform (Novogene, Beijing, China). The reference genome was GDDH13v1.1. The primers used for ChIP–qPCR were designed to amplify regions in the promoter sequences of *MdGORK1* and *MdNHX2* (Table S3).

### Electrophoretic mobility shift assay (EMSA)

The full‐length *MdWRKY53* coding sequence was cloned into the pGEX6p‐1 vector to generate the GST–MdWRKY53 fusion protein, which was produced in *E. coli* (BL21) and purified using glutathione Sepharose 4B (GE) as described previously (Zheng *et al*., 2018). For EMSA, the *MdGORK1* and *MdNHX2* promoter fragments containing the W‐box were synthesized using a biotin‐labelled oligonucleotide or unlabeled oligonucleotide (to be used as a competitor). EMSA was conducted using a LightShift chemiluminescence EMSA kit (Thermo Scientific) according to the manufacturer's instructions.

### Dual‐luciferase (dual‐LUC) assay

The *MdNHX2* and *MdGORK1* promoters were amplified by PCR from apple GL3 genomic DNA and cloned upstream of the firefly luciferase gene (*LUC*) in pGreenII 0800‐LUC as reporter constructs, and the coding sequence of *MdWRKY53* was cloned into pGreenII 62‐SK as the effector construct. The constructs were separately introduced into Agrobacterium strain GV3101. The leaves of one‐month‐old *N. benthamiana* plants were infiltrated with pairs of Agrobactreium cultures harbouring *ProMdNHX2*:*LUC* + SK, *ProMdNHX2*:*LUC* + *MdWRKY53*‐SK, *ProMdGORK1*:*LUC* + SK, and *ProMdGORK1*:*LUC* + *MdWRKY53*‐SK for transient expression. The LUC activities were detected 3 days after infiltration with reagents from the Dual Glow assay for Firefly LUC and *Renilla* luciferase (REN) (Targeting Systems, San Diego, CA, USA) on a luminometer as described by Zheng *et al*. (2022). The ratio of LUC to REN activity was used to quantify relative LUC activity. Primers used in the LUC tests are listed in Table S3.

### Statistical analysis

The data were subjected to analysis of variance (ANOVA), followed by Tukey's honestly significant difference (HSD) test. Statistically significant differences at *P* < 0.05 are indicated. Statistical computations were carried out using SPSS software (IBM, Armonk, NY, USA).

## Author contributions

X. Z. planned and designed the research. Z. S., D. G., J. L., T. W., Y. T., C. M., X. L. and C. W. performed experiments, conducted fieldwork and analysed data etc. X. Z., J. L. and D. G. wrote the article.

## Conflict of interest

The authors declare that the research was conducted in the absence of any commercial or financial relationships that could be construed as a potential conflict of interest.

## Supporting information


**Figure S1** Melatonin (MT) alleviates KCl stress in apple rootstocks. The wilting rate (a), fresh weight (b) and dry weight (c) of seven kinds of apple rootstocks treated by 50 mm KCl stress for 20 days. The phenotype (d), wilting rate (e), fresh weight (f), dry weight (g) of *Qingzhen 1*, *Malus hupenensis*, and *M26* treated by 50 mm KCl and 100 μm MT for 20 days. The net K^+^ flux (h) and K^+^ distribution (i) in roots of *Qingzhen 1*, *Malus hupenensis*, and *M26* plants treated with KCl stress and exogenous 100 μm MT for 6 h. Data represent the means ± SD of triplicate experiments. Different lowercase letters indicate significant differences according to Tukey's HSD (*P* < 0.05).
**Figure S2** Effects of exogenous MT on oxidative damage and osmotic stress under KCl stress in *M. hupehensis* seedlings. ROS staining of roots (a) and O_2_·^−^ and H_2_O_2_ staining of leaves (b) in *M. hupehensis* seedlings after KCl stress and MT treatment for 30 days. Effects of exogenous MT on ROS content in roots (c), MDA content (d), SOD activity (e), POD activity (f), CAT activity (g), electrolyte leakage (h), proline content (i) and soluble sugar content (j) under KCl stress in *M. hupehensis* seedlings. Data represent the means ± SD of triplicate experiments. Different lowercase letters indicate significant differences according to Tukey's HSD (*P* < 0.05).
**Figure S3** Effects of exogenous MT on mineral elements under KCl stress. Effects of MT on macronutrient content (a), micronutrient content (b) and K:Na ratio (c) under KCl stress in *M*. *hupehensis* seedlings. Data represent the means ± SD of triplicate experiments. Different lowercase letters indicate significant differences according to Tukey's HSD (*P* < 0.05).
**Figure S4** Relative expression of *MdWRKY53*, *MdGORK1*, and *MdNHX2* under KCl stress and MT treatment in *Qingzhen 1*, *Malus hupenensis*, and M26. (a) Gene ontology (GO) enrichment and (b) Kyoto encyclopedia of genes and genomes (KEGG) analysis of differentially expressed genes in *Malus hupenensis* seedlings treated with KCl stress and exogenous MT. Relative expression of *MdWRKY53* (c), *MdGORK1* (d), and *MdNHX2* (e) in leaves and roots of *Malus hupenensis* under KCl stress and MT treatment. Relative expression of *MdWRKY53* (f), *MdGORK1* (g), and *MdNHX2* (h) in *Qingzhen 1*, *Malus hupenensis*, and M26 under KCl stress and MT treatment. Data represent the means ± SD of triplicate experiments. Different lowercase letters indicate significant differences according to Tukey's HSD (*P* < 0.05).
**Figure S5** The phenotype of *AtWRKY41* transgenic *Arabidopsis* under KCl stress and MT treatment. Relative expression of *MdWRKY53* in transgenic and wild type apple plants (a) and callus (b). (c) Relative expression level of *AtWRKY41* in OE *AtWRKY41*, Col‐0, and *wrky41 Arabidopsis*. Phenotype (d) and wilting rate (e) of OE *AtWRKY41*, Col‐0, and *wrky41 Arabidopsis* treated by 50 mm KCl for 30 days. Phenotype (f), wilting rate (g), and fresh weight (h) of Col‐0 and *wrky41 Arabidopsis* under KCl stress and MT treatment. Data represent the means ± SD of triplicate experiments. Different lowercase letters indicate significant differences according to Tukey's HSD (*P* < 0.05).
**Figure S6** Phenotype of OEC *MdWRKY53*, OEC *MdWRKY53* + RNAi *MdGORK1*, OEC *MdWRKY53* + RNAi *MdNHX2* transgenic apple callus under KCl stress. Phenotype (a) and browning rate (c) of OEC *MdWRKY53*, OEC *MdWRKY53* + RNAi *MdGORK1*, OEC *MdWRKY53* + RNAi *MdNHX2* transgenic and wild type apple callus under KCl stress for 30 days. (b) Relative expression of *MdWRKY53*, *MdGORK1*, and *MdNHX2* in OEC *MdWRKY53*, OEC *MdWRKY53* + RNAi *MdGORK1*, OEC *MdWRKY53* + RNAi *MdNHX2* transgenic apple callus. (d) Relative expression of *MdGORK1* in transgenic apple plants overexpressing *MdGORK1* and wild type plants. (e) Net K^+^ flux of OE *MdNHX2* transgenic lines and wild type apple plants under KCl stress. Data represent the means ± SD of triplicate experiments. Different lowercase letters indicate significant differences according to Tukey's HSD (*P* < 0.05).
**Figure S6** Phenotype of OEC *MdWRKY53*, OEC *MdWRKY53* + RNAi *MdGORK1*, OEC *MdWRKY53* + RNAi *MdNHX2* transgenic apple callus under KCl stress. Phenotype (a) and browning rate (c) of OEC *MdWRKY53*, OEC *MdWRKY53* + RNAi *MdGORK1*, OEC *MdWRKY53* + RNAi *MdNHX2* transgenic and wild type apple callus under KCl stress for 30 days. (b) Relative expression of *MdWRKY53*, *MdGORK1*, and *MdNHX2* in OEC *MdWRKY53*, OEC *MdWRKY53* + RNAi *MdGORK1*, OEC *MdWRKY53* + RNAi *MdNHX2* transgenic apple callus. (d) Relative expression of *MdGORK1* in transgenic apple plants overexpressing *MdGORK1* and wild type plants. (e) Net K^+^ flux of OE *MdNHX2* transgenic lines and wild type apple plants under KCl stress. Data represent the means ± SD of triplicate experiments. Different lowercase letters indicate significant differences according to Tukey's HSD (*P* < 0.05).
**Table S1** Statistical table of sequencing data.
**Table S2** Statistical table of sequence alignment results between sample sequencing data and selected reference genomes.
**Table S3** The primers used for cloning, vector construction, qRT‐PCR and EMSA.
