# Supplementary material for: Melatonin enhances KCl salinity tolerance by maintaining K+ homeostasis in Malus hupehensis
Source: Plant Biotechnol J. 2023 Jul 19;21(11):2273–90. doi: 10.1111/pbi.14129 (PMC10579713; doi:10.1111/pbi.14129)
Supplement: Supplementary file 1 — Figure S1 Melatonin (MT) alleviates KCl stress in apple rootstocks. The wilting rate (a), fresh weight (b) and dry weight (c) of seven kinds of apple rootstocks treated by 50 mm KCl stress for 20 days. The phenotype (d), wilting rate (e), fresh weight (f), dry weight (g) of Qingzhen 1, Malus hupenensis, and M26 treated by 50 mm KCl and 100 μm MT for 20 days. The net K+ flux (h) and K+ distribution (i) in roots of Qingzhen 1, Malus hupenensis, and M26 plants treated with KCl stress and exogenous 100 μm MT for 6 h. Data represent the means ± SD of triplicate experiments. Different lowercase letters indicate significant differences according to Tukey's HSD (P < 0.05). Figure S2 Effects of exogenous MT on oxidative damage and osmotic stress under KCl stress in M. hupehensis seedlings. ROS staining of roots (a) and O2·− and H2O2 staining of leaves (b) in M. hupehensis seedlings after KCl stress and MT treatment for 30 days. Effects of exogenous MT on ROS content in roots (c), MDA content (d), SOD activity (e), POD activity (f), CAT activity (g), electrolyte leakage (h), proline content (i) and soluble sugar content (j) under KCl stress in M. hupehensis seedlings. Data represent the means ± SD of triplicate experiments. Different lowercase letters indicate significant differences according to Tukey's HSD (P < 0.05). Figure S3 Effects of exogenous MT on mineral elements under KCl stress. Effects of MT on macronutrient content (a), micronutrient content (b) and K:Na ratio (c) under KCl stress in M. hupehensis seedlings. Data represent the means ± SD of triplicate experiments. Different lowercase letters indicate significant differences according to Tukey's HSD (P < 0.05). Figure S4 Relative expression of MdWRKY53, MdGORK1, and MdNHX2 under KCl stress and MT treatment in Qingzhen 1, Malus hupenensis, and M26. (a) Gene ontology (GO) enrichment and (b) Kyoto encyclopedia of genes and genomes (KEGG) analysis of differentially expressed genes in Malus hupenensis seedlings treate [file PBI-21-2273-s001.docx]

**Supplemental Figures**

**Figure S1**

**
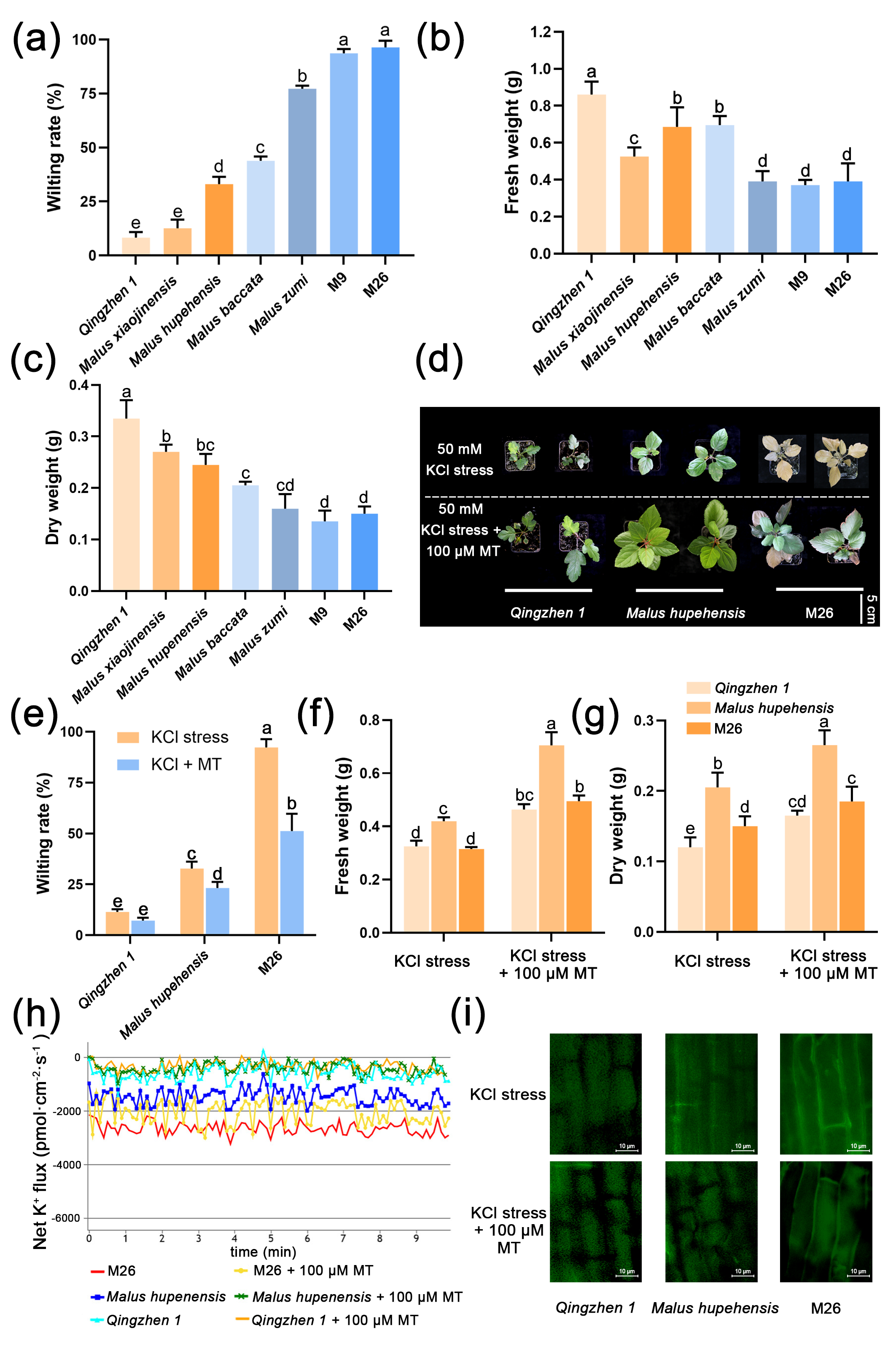
**

**Figure S1** Melatonin (MT) alleviates KCl stress in apple rootstocks. The wilting rate (a), fresh weight (b) and dry weight (c) of seven kinds of apple rootstocks treated by 50 mM KCl stress for 20 days. The phenotype (d), wilting rate (e), fresh weight (f), dry weight (g) of *Qingzhen 1*, *Malus hupenensis*, and *M26* treated by 50 mM KCl and 100 μM MT for 20 days. The net K^+^ flux (h) and K^+^ distribution (i) in roots of *Qingzhen 1*, *Malus hupenensis*, and *M26* plants treated with KCl stress and exogenous 100 μM MT for 6 h. Data represent the means ± SD of triplicate experiments. Different lowercase letters indicate significant differences according to Tukey’s HSD (*P* < 0.05).

**Figure S2**


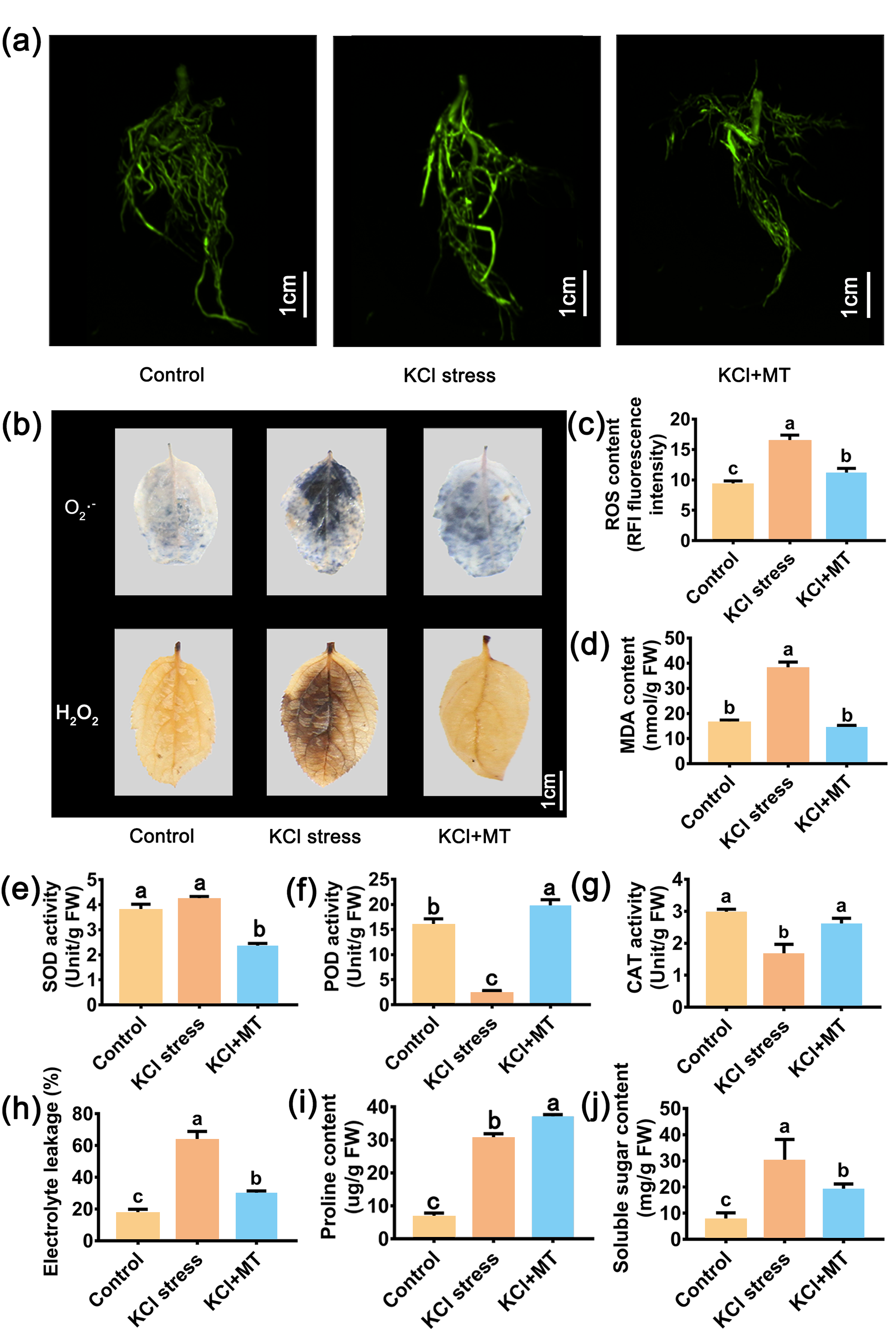


**Figure S2** Effects of exogenous MT on oxidative damage and osmotic stress under KCl stress in *M. hupehensis* seedlings. ROS staining of roots (a) and O_2_·^−^ and H_2_O_2_ staining of leaves (b) in *M. hupehensis* seedlings after KCl stress and MT treatment for 30 days. Effects of exogenous MT on ROS content in roots (c), MDA content (d), SOD activity (e), POD activity (f), CAT activity (g), electrolyte leakage (h), proline content (i) and soluble sugar content (j) under KCl stress in *M. hupehensis* seedlings. Data represent the means ± SD of triplicate experiments. Different lowercase letters indicate significant differences according to Tukey’s HSD (*P* < 0.05).

**Figure S3**


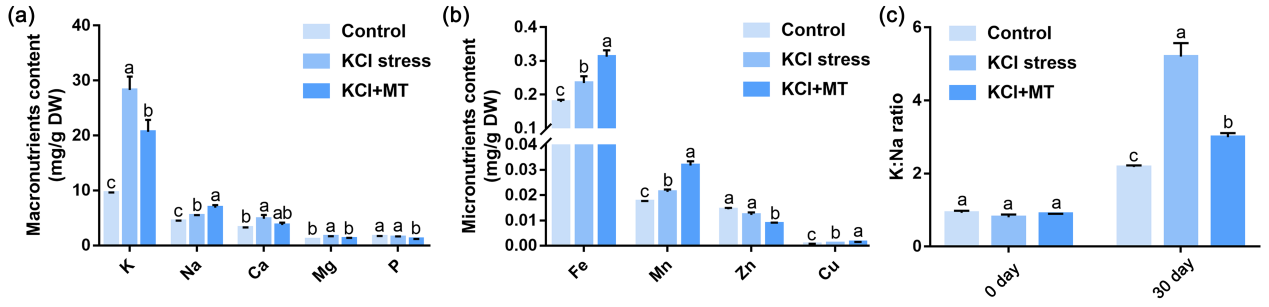


**Figure S3** Effects of exogenous MT on mineral elements under KCl stress. Effects of MT on macronutrient content (a), micronutrient content (b) and K:Na ratio (c) under KCl stress in *M*. *hupehensis* seedlings. Data represent the means ± SD of triplicate experiments. Different lowercase letters indicate significant differences according to Tukey’s HSD (*P* < 0.05).

**Figure S4**


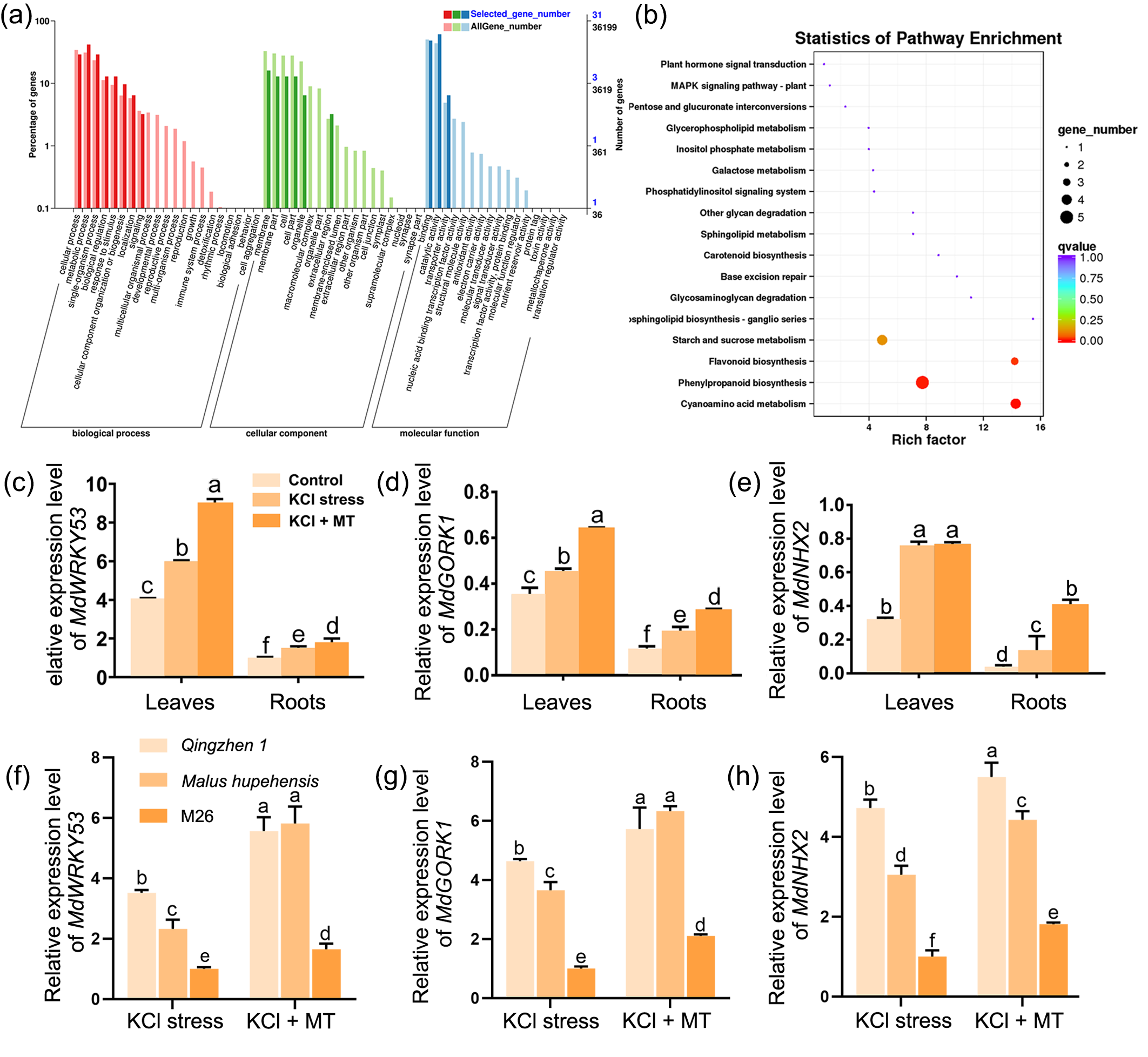


**Figure S4** Relative expression of *MdWRKY53*, *MdGORK1*, and *MdNHX2* under KCl stress and MT treatment in *Qingzhen 1*, *Malus hupenensis*, and M26. (a) Gene ontology (GO) enrichment and (b) Kyoto encyclopedia of genes and genomes (KEGG) analysis of differentially expressed genes in *Malus hupenensis* seedlings treated with KCl stress and exogenous MT. Relative expression of *MdWRKY53* (c), *MdGORK1* (d), and *MdNHX2* (e) in leaves and roots of *Malus hupenensis* under KCl stress and MT treatment. Relative expression of *MdWRKY53* (f), *MdGORK1* (g), and *MdNHX2* (h) in *Qingzhen 1*, *Malus hupenensis*, and M26 under KCl stress and MT treatment. Data represent the means ± SD of triplicate experiments. Different lowercase letters indicate significant differences according to Tukey’s HSD (*P* < 0.05).

**Figure S5**


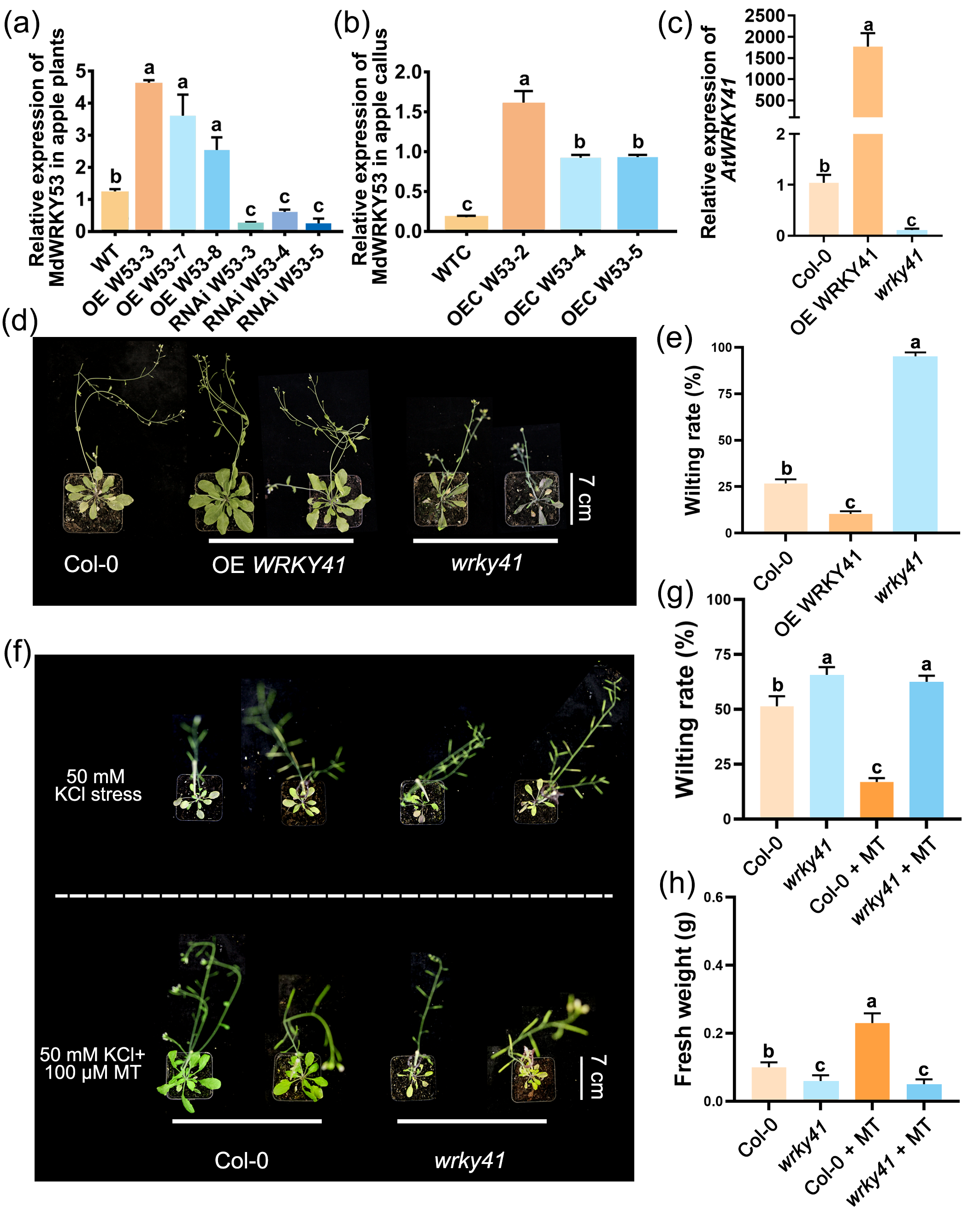


**Figure S5** The phenotype of *AtWRKY41* transgenic *Arabidopsis* under KCl stress and MT treatment. Relative expression of *MdWRKY53* in transgenic and wild type apple plants (a) and callus (b). (c) Relative expression level of *AtWRKY41* in OE *AtWRKY41*, Col-0, and *wrky41* *Arabidopsis*. Phenotype (d) and wilting rate (e) of OE *AtWRKY41*, Col-0, and *wrky41* *Arabidopsis* treated by 50 mM KCl for 30 days. Phenotype (f), wilting rate (g), and fresh weight (h) of Col-0 and *wrky41* *Arabidopsis* under KCl stress and MT treatment. Data represent the means ± SD of triplicate experiments. Different lowercase letters indicate significant differences according to Tukey’s HSD (*P* < 0.05).

**Figure S6**

**
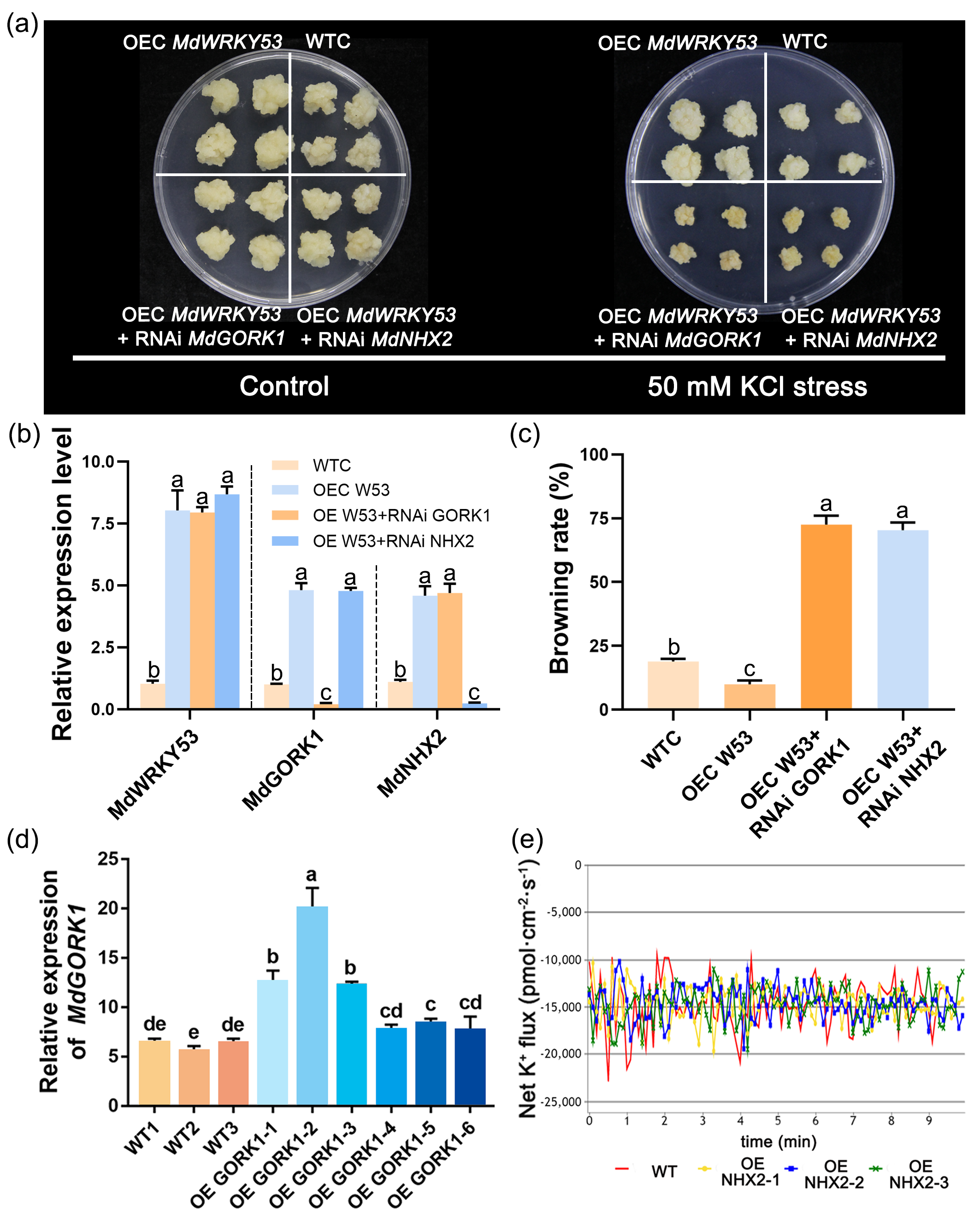
**

**Figure S6** Phenotype of OEC *MdWRKY53*, OEC *MdWRKY53* + RNAi *MdGORK1*, OEC *MdWRKY53* + RNAi *MdNHX2* transgenic apple callus under KCl stress. Phenotype (a) and browning rate (c) of OEC *MdWRKY53*, OEC *MdWRKY53* + RNAi *MdGORK1*, OEC *MdWRKY53* + RNAi *MdNHX2* transgenic and wild type apple callus under KCl stress for 30 days. (b) Relative expression of *MdWRKY53*, *MdGORK1*, and *MdNHX2* in OEC *MdWRKY53*, OEC *MdWRKY53* + RNAi *MdGORK1*, OEC *MdWRKY53* + RNAi *MdNHX2* transgenic apple callus. (d) Relative expression of *MdGORK1* in transgenic apple plants overexpressing *MdGORK1* and wild type plants. (e) Net K^+^ flux of OE *MdNHX2* transgenic lines and wild type apple plants under KCl stress. Data represent the means ± SD of triplicate experiments. Different lowercase letters indicate significant differences according to Tukey’s HSD (*P* < 0.05).

**Supplemental tables**

**Table S1 Statistical table of sequencing data**

| Samples | Clean reads | Clean bases | GC Content | %≥Q30 |
| --- | --- | --- | --- | --- |
| CKL-1 | 23,758,186 | 7,085,105,792 | 47.38% | 92.57% |
| CKL-2 | 23,972,530 | 7,149,888,124 | 47.49% | 94.93% |
| CKL-3 | 21,763,408 | 6,493,695,080 | 47.52% | 94.75% |
| CKR-1 | 20,682,357 | 6,183,208,376 | 48.03% | 91.74% |
| CKR-2 | 20,932,706 | 6,256,617,340 | 48.09% | 94.92% |
| CKR-3 | 21,540,534 | 6,442,654,920 | 48.14% | 94.83% |
| KCLL-1 | 22,255,311 | 6,633,753,738 | 47.49% | 92.03% |
| KCLL-2 | 27,287,155 | 8,137,940,190 | 47.48% | 94.78% |
| KCLL-3 | 26,339,551 | 7,839,723,918 | 47.41% | 94.52% |
| KCLR-1 | 21,007,338 | 6,281,600,116 | 49.28% | 90.83% |
| KCLR-2 | 25,992,461 | 7,772,239,756 | 49.36% | 94.79% |
| KCLR-3 | 19,637,392 | 5,866,254,616 | 49.22% | 94.76% |
| MTL-1 | 25,541,845 | 7,619,262,928 | 47.26% | 92.50% |
| MTL-2 | 21,202,571 | 6,319,727,808 | 47.49% | 94.99% |
| MTL-3 | 21,842,021 | 6,512,956,208 | 47.35% | 94.98% |
| MTR-1 | 23,780,981 | 7,106,528,952 | 47.98% | 93.16% |
| MTR-2 | 19,385,948 | 5,796,111,242 | 48.11% | 94.87% |
| MTR-3 | 21,135,724 | 6,318,730,166 | 48.15% | 95.04% |

**Table S2 Statistical table of sequence alignment results between sample sequencing data and selected reference genomes**

| Samples | Total Reads | Mapped Reads | Uniq Mapped Reads |
| --- | --- | --- | --- |
| CKL-1 | 47,516,372 | 40,720,555 （85.70%） | 37,897,617 （79.76%） |
| CKL-2 | 47,945,060 | 41,266,723 （86.07%） | 38,354,610 （80.00%） |
| CKL-3 | 43,526,816 | 37,351,798 （85.81%） | 34,720,156 （79.77%） |
| CKR-1 | 41,364,714 | 26,990,097 （65.25%） | 26,264,555 （63.50%） |
| CKR-2 | 41,865,412 | 27,257,768 （65.11%） | 26,544,977 （63.41%） |
| CKR-3 | 43,081,068 | 28,196,783 （65.45%） | 27,454,575 （63.73%） |
| KCLL-1 | 44,510,622 | 37,676,042 （84.65%） | 34,729,018 （78.02%） |
| KCLL-2 | 54,574,310 | 46,419,098 （85.06%） | 42,753,805 （78.34%） |
| KCLL-3 | 52,679,102 | 44,844,390 （85.13%） | 41,305,303 （78.41%） |
| KCLR-1 | 42,014,676 | 26,805,459 （63.80%） | 26,130,199 （62.19%） |
| KCLR-2 | 51,984,922 | 33,396,258 （64.24%） | 32,521,696 （62.56%） |
| KCLR-3 | 39,274,784 | 25,249,474 （64.29%） | 24,605,332 （62.65%） |
| MTL-1 | 51,083,690 | 43,908,913 （85.95%） | 40,685,414 （79.64%） |
| MTL-2 | 42,405,142 | 36,244,379 （85.47%） | 33,461,586 （78.91%） |
| MTL-3 | 43,684,042 | 37,445,989 （85.72%） | 34,618,004 （79.25%） |
| MTR-1 | 47,561,962 | 38,399,415 （80.74%） | 37,237,316 （78.29%） |
| MTR-2 | 38,771,896 | 31,407,246 （81.01%） | 30,467,812 （78.58%） |
| MTR-3 | 42,271,448 | 34,247,404 （81.02%） | 33,197,923 （78.54%） |

**Table S3 The primers used for cloning, vector construction, qRT-PCR and EMSA**

| Primer name | Forward primer | Reverse primer | Vector |
| --- | --- | --- | --- |
| MdActin | CTTCAATGTGCCTGCCATGTAT | AATTTCCCGTTCAGCAGTAGTG |  |
| Cloning the CDS of  MdWRKY53 | ATGGATTCTGGTAAGAGCTG | TTATAAGAAAAATCCTGGG |  |
| qMdWRKY53 | CTGAGAATGGGATTGAAGGAC | ATTGTGTCGTTTTCGTTCTTGC |  |
| MdWRKY53-GFP | ACTAGTATGGATTCTGGTAAGAGCTG | GGCGCGTTATAAGAAAAATCCTGGG | *pMDC83* |
| MdWRKY53-BD | GAATCCATGGATTCTGGTAAGAGCTG | CCTAGGCTTATAAGAAAAATCCTGGG | *pGBKT7* |
| MdWRKY53-pBI121 | GGATCCATGGATTCTGGTAAGAGCTG | GGGCCCTTATAAGAAAAATCCTGGG | *pBI121* |
| MdWRKY53-GST | GGATCCATGGATTCTGGTAAGAGCTG | GAGCTCTTATAAGAAAAATCCTGGG | *pGEX6p-1* |
| MdWRKY53-SK | CGCGGTGGCGGCCGCTCTAGAATGGATTCTGGTAAGAGCTGGG | TTCCTGCAGCCCGGGGGATCCTAAGAAAAATCCTGGGGAGTCAA | *pGreenII 62-SK* |
| Cloning the CDS of  MdGORK1 | ATGCATGGAGCTGAAAGGAG | TTAACCAGTTTCTTGAGAAT |  |
| qMdGORK1 | ATGATGATTATGAGGAGGAGCA | GGAGTAGTAGTAAGAGTGGTGGAGT |  |
| MdGORK1-ChIP-qPCR | GTGGTCGACAATGATTCAGA | CTAGTTAGAAAGTTCCCACTT |  |
| MdGORK1-GFP | ACTAGTATGCATGGAGCTGAAAGGAG | GGCGCGCCAACCAGTTTCTTGAGAATGAT | *pMDC83* |
| MdGORK1-pBI121 | GAGAACACGGGGGACTCTAGAATGCATGGAGCTGAAAGGAGAA | AAGGGACTGACCACCCGGGGATTAACCAGTTTCTTGAGAATGATCG | *pBI121* |
| Cloning the Promoter of the MdGORK1 | CCATCGACTCAAGAGTCA | ATCTTCCTCAGATCGACCTC |  |
| ProMdGORK1-LUC | TATAGGGCGAATTGGGTACCGACTGCTTAATTATGCCAATCCATT | TCTCCACCGCGGTGGCGGCCGCTTTTTATCTTCCTCAGATCGACCTC | *pGreenII 0800-LUC* |
| For EMSA of MdGORK1 | TTATGTCACCTTATGTTTTTGACACAATTTTTATAACGTTGACACAG | CTGTGTCAACGTTATAAAAATTGTGTCAAAAACATAAGGTGACATAA |  |
| Cloning the CDS of  MdNHX2 | ACTTTTCAGCTTCCCAGAAACGA | CTTGTCAATGTCCAGGGTATCCA |  |
| qMdNHX2 | GCTTATGCGTGGCTCTGTTTC | CGGGTCCTATGTTTGCCTCTG |  |
| MdNHX2-ChIP-qPCR | TAATTCTCTTCTGTTTTGCA | GAGAGCAATTCAGTTCAA |  |
| MdNHX2-GFP | ACTAGTATGGCGGTCGATGCGAG | GGCGCGCCAATGCCATTGATTTCCA | *pMDC83* |
| MdNHX2-pBI121 | TCTAGAATGGCGGTCGATGCGAG | GGATCCATGCCATTGATTTCCA | *pBI121* |
| Cloning the Promoter of the MdNHX2 | TCTTTTGAGCGACAGAAGGGAG | AGCAATTCAGTTCAAGTTCCAC |  |
| ProMdNHX2-LUC | TATAGGGCGAATTGGGTACCTCTTTTGAGCGACAGAAGGGAG | TCTCCACCGCGGTGGCGGCCGCAGCAATTCAGTTCAAGTTCCACTTG | pGreenII 0800-LUC |
| For EMSA of MdNHX2 | TGCACACGTCTTTCGAAGTTGACCAGATCAGTGTGCTTTGA | TCAAAGCACACTGATCTGGTCAACTTCGAAAGACGTGTGCA |  |
